# Identification and characterization of a novel gene involved in glandular trichome development in *Nepeta tenuifolia*

**DOI:** 10.3389/fpls.2022.936244

**Published:** 2022-07-29

**Authors:** Peina Zhou, Jingjie Dang, Zunrui Shi, Yongfang Shao, Mengru Sang, Shilin Dai, Wei Yue, Chanchan Liu, Qinan Wu

**Affiliations:** ^1^College of Pharmacy, Nanjing University of Chinese Medicine, Nanjing, China; ^2^Collaborative Innovation Center of Chinese Medicinal Resources Industrialization, Nanjing, China; ^3^National and Local Collaborative Engineering Center of Chinese Medicinal Resources Industrialization and Formulae Innovative Medicine, Nanjing, China

**Keywords:** glandular trichome development, *Nepeta tenuifolia*, VIGS, transcriptomic (RNA-seq), metabolome

## Abstract

*Nepeta tenuifolia* is a medicinal plant rich in terpenoids and flavonoids with antiviral, immunoregulatory, and anti-inflammatory activities. The peltate glandular trichome (PGT) is a multicellular structure considered to be the primary storage organ for monoterpenes; it may serve as an ideal model for studying cell differentiation and the development of glandular trichomes (GTs). The genes that regulate the development of GTs have not yet been well studied. In this study, we identified NtMIXTA1, a GT development-associated gene from the R2R3 MYB SBG9 family. *NtMIXTA1* overexpression in tobacco resulted in the production of longer and denser GTs. Virus-induced gene silencing of *NtMIXTA1* resulted in lower PGT density, a significant reduction in monoterpene concentration, and the decreased expression of genes related to monoterpene biosynthesis. Comparative transcriptome and widely targeted metabolic analyses revealed that silencing *NtMIXTA1* significantly influenced the expression of genes, and the production of metabolites involved in the biosynthesis of terpenoids, flavonoids, and lipids. This study provides a solid foundation describing a mechanism underlying the regulation of GT development. In addition, this study further deepens our understanding of the regulatory networks involved in GT development and GT development-associated metabolite flux, as well as provides valuable reference data for studying plants with a high medicinal value without genetic transformation.

## Introduction

Trichomes are specialized organs that originate from the epidermis and are made up of single or multiple cells. Trichomes are classified into two types based on their potential for secretion: glandular trichomes (GTs) and non-glandular trichomes (NGTs) (Werker, 2000). GTs are regarded as cell biofactories because they can synthesize and accumulate a variety of valuable metabolites, including polysaccharides, terpenoids (such as artemisinin and sclareol), methyl ketones, and acyl sugars (Tissier, 2012; Wang, 2015). These metabolites serve as chemical barriers that protect plants from herbivorous insects and pathogens. They are also widely used as medicines, spices, food additives, and pesticides (Duke et al., 2000). Recent research has focused on the molecular mechanisms underlying trichome formation and development. The specific regulators of these processes have been characterized in *Artemisia annua*, tomato, and cucumber (Chalvin et al., 2020). The members of the R2R3 MYB family, which is the largest MYB subfamily, have diverse functions in regulating secondary metabolism, cell development, responses to multiple stressors, and hormonal signal transport (Stracke et al., 2001; Feller et al., 2011). The R2R3 MYB family is divided into 22 subgroups based on sequence and functional similarities (Dubos et al., 2010). Several studies have implied that members from subgroup 9 of the R2R3 MYB family (R2R3 SBG9) are important for epidermal differentiation, particularly that associated with trichomes (Brockington et al., 2013). They are represented by *AtMYB16, AtMYB106*, and *AtMYB17* in *Arabidopsis thaliana, AmMIXTA-LIKE 1, AmMIXTA-LIKE 2* in *Antirrhinum majus, GhMYB25* in *Gossypium hirsuta, AaMIXTA1* in *A. annua*, and *SlMX1* in tomato, which are involved in trichome formation and development (Perez-Rodriguez et al., 2005; Walford et al., 2011; Ewas et al., 2016; Shi et al., 2018).

*Nepeta tenuifolia* Briq. is a member of the Lamiaceae family. It has an intense aroma and has been widely used as a traditional medicine in Asia (Liu et al., 2018). Due to its excellent antiviral, immunoregulatory, and anti-inflammatory properties, *N. tenuifolia* has been included as a component of the Chinese medicine Jingfang Baidu powder for the treatment of severe acute respiratory syndrome (SARS) and coronavirus disease 2019 (COVID-19) (Zhang et al., 2003; He et al., 2013; Feng Q. et al., 2020). Previous phytochemical studies have reported that, among the numerous compounds found in *N. tenuifolia*, volatile oils, primarily monoterpenes, exhibit the most significant biological activity. *N. tenuifolia* leaves, stems, and spikes contain three forms of GTs: peltate GTs (PGTs), where monoterpenes are specifically synthesized, capitate GTs, and digitiform GTs (Liu et al., 2018). There is a strong correlation between the number of PGTs and the volatile oil content. In addition, gene regulation of monoterpene production in PGTs has been elucidated (Liu et al., 2018, 2021). Nonetheless, the molecular mechanisms underlying the development of PGTs remain unknown.

Previous research has demonstrated that GT initiation may be related to the production of its specialized metabolites. For example, *SlMYC1* in tomatoes regulates the initiation of the type VI secretion system, which also modulates terpene biosynthesis (Xu et al., 2019). In *A. annua*, artemisinin content is proportional to GT density. Repressing the expression of *AaMIXTA1*, a positive regulator of GTs, decreases the number of GSTs, artemisinin content, and cuticle deposition (Shi et al., 2018). GT initiation is also implicated in cuticle biosynthesis; however, the relationship between trichome formation and cutin and wax biosynthesis remains unknown. Intriguingly, in addition to terpenoids, GT initiation also affects flavonoid biosynthesis, even though these two pathways operate independently (Sugimoto et al., 2021). The tomato mutant *odorless-2 (od-2)* exhibits defects in the development and density of GTs, as well as a disruption in the production of terpenes and flavonoids (Kang et al., 2010). *AaTAR2* of *A. annua* positively regulates trichome development and biosynthesis of artemisinin and flavonoids (Zhou et al., 2020). Researches have demonstrated that members of the MYB family play a coordinated metabolic role in flavonoid and terpenoid biosynthesis pathways (Bedon et al., 2010; Zvi et al., 2012). However, the gene regulatory networks involved in GT formation and metabolite variations remain unclear.

Gene characterization and genetic research in plants are typically inseparable from the development of transgenic systems. However, in the field of medicinal plants, few genetic transformation techniques are available, and their establishment requires lengthy timelines, posing challenges to the molecular property research. Recently, virus-induced gene silencing (VIGS) has been widely used for gene functional studies. The method is available and effective for no stable system of genetic transformation (Unver and Budak, 2009; Courdavault et al., 2020; Zang et al., 2021).

In this study, we identified and characterized the gene *NtMIXTA1*, which is a member of the R2R3 MYB SBG9 transcription factor family. This *NtMIXTA1*-overexpressed tobacco showed an increase in GT length and density. Silencing *NtMIXTA1* expression resulted in the decrease in PGT density and menthane monoterpenoids. We also investigated the expression of related genes and variation in the levels of metabolites using the transcriptomic and widely targeted metabolic analysis of *NtMIXTA1*-silenced plants. The findings demonstrated that *NtMIXTA1* may be a positive regulator of PGT initiation and that the biosynthesis of terpenoids, flavonoids, and lipids was significantly affected. Our findings provide valuable information for future research on the initiation of trichome formation and the associated metabolic pathway.

## Results

### Isolation and characterization of *NtMIXTA1*

**Figure 1:**
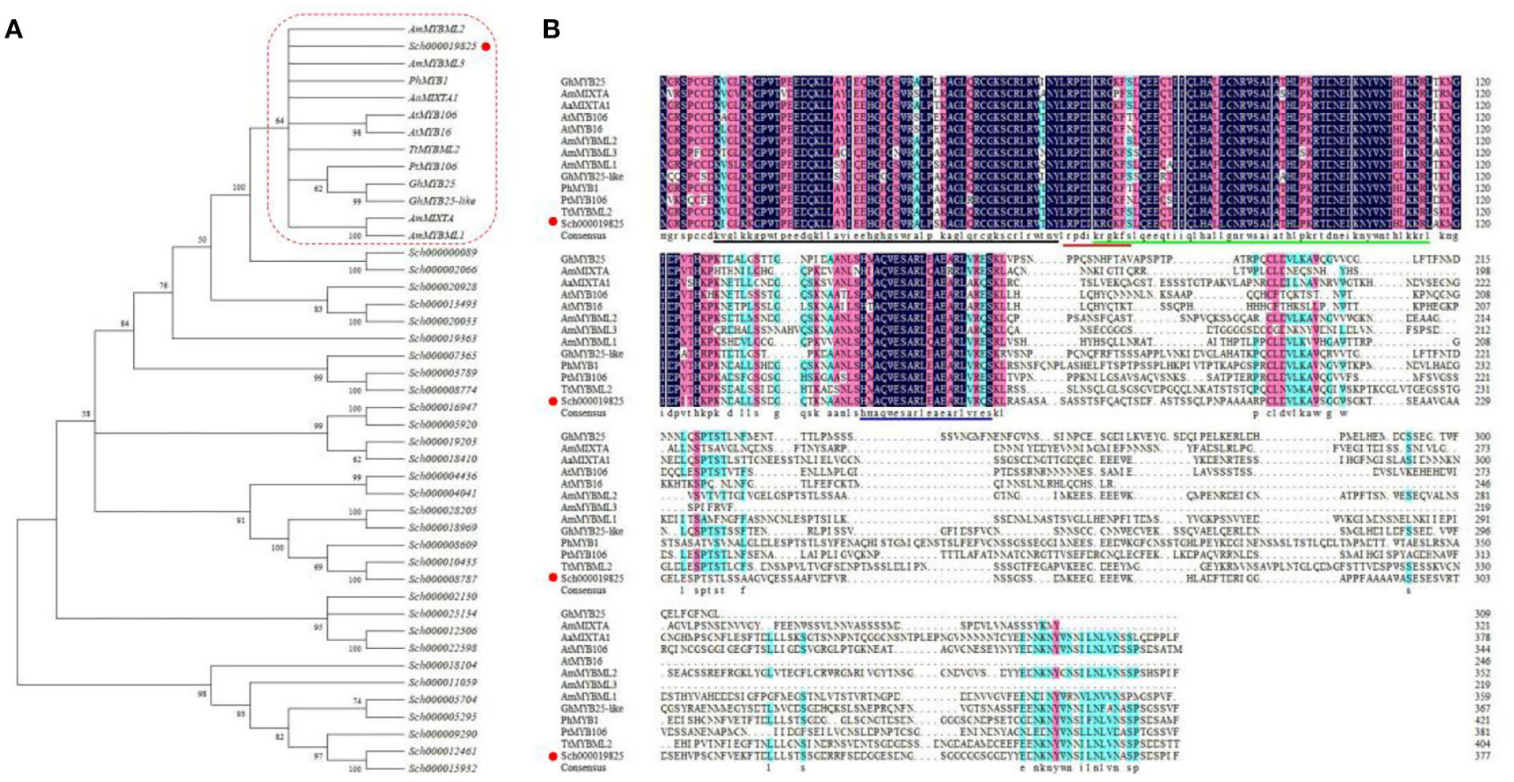
Sequence analysis of NtMIXTA1. **(A)** ML (Maximum-likelihood) tree of R2R3 SBG9 proteins. *Arabidopsis thaliana* (At), *Petunia hybrida* (Ph), *Artemisia annua* (Aa), *Gossypium hirsutum* (Gh), *Thalictrum thalictroides* (Tt), *Antirrhinum majus* (Am), and *Populus trichopoda* (Pt). **(B)** Alignment of the protein sequences of NtMIXTA1 and reported R2R3 SBG9 proteins. The R2 and R3 domain characteristics are shown with back and green lines; the conserved domain of subgroup 9 is blue; the red line indicates the putative nuclear localization signal (NLS).

To identify the genes belonging to the R2R3 SBG9, 32 sequences were screened on the basis of the family domains and characterized protein sequences of members from the R2R3 SBG9 (from the NCBI) (Brockington et al., 2013). Phylogenetic analysis of the 32 candidate genes revealed that only *Sch000019825* clustered with sequences of the SBG9 R2R3 MYB members. The function of SBG9 R2R3 MYB members in regulating epidermal cell differentiation has been elucidated, especially in trichomes (Baumann et al., 2007) (Figure 1A). The multiple sequence alignment of these sequences revealed that Sch000019825 shared a common region “HxAQWESARLxAExRLxRxS” near the amino (N) terminal and a highly conserved R2R3-MYB domain close to the carboxy (C) terminal, demonstrating that it can be assigned to the R2R3 SBG9 (Zhang et al., 2016; Shi et al., 2018; Qin et al., 2021) (Figure 1B). The functions in trichomes and epidermal development of AmMYBML2, AmMYBML3, PhMYB1, and AaMIXTA1 have been characterized (Baumann et al., 2007; Jaffe et al., 2007; Shi et al., 2018), and Sch000019825 is similar to above proteins. Based on the aforementioned information, *Sch000019825* was named *NtMIXTA1* and was analyzed further. The open reading frame (ORF) of *NtMIXTA1* is 1,134 bp in size, encoding 377 amino acids. Based on ExPASy (https://www.expasy.org/) analysis, the formula of the encoded protein is C_1767_H_2752_N_518_O_575_S_10_; the theoretical isoelectric point and molecular weight of the protein are 6.17 and 44.773 kDa, respectively. Furthermore, the protein may be unstable, with an instability index of 51.19.

### Expression profiles and subcellular localization of *NtMIXTA1*

**Figure 2:**
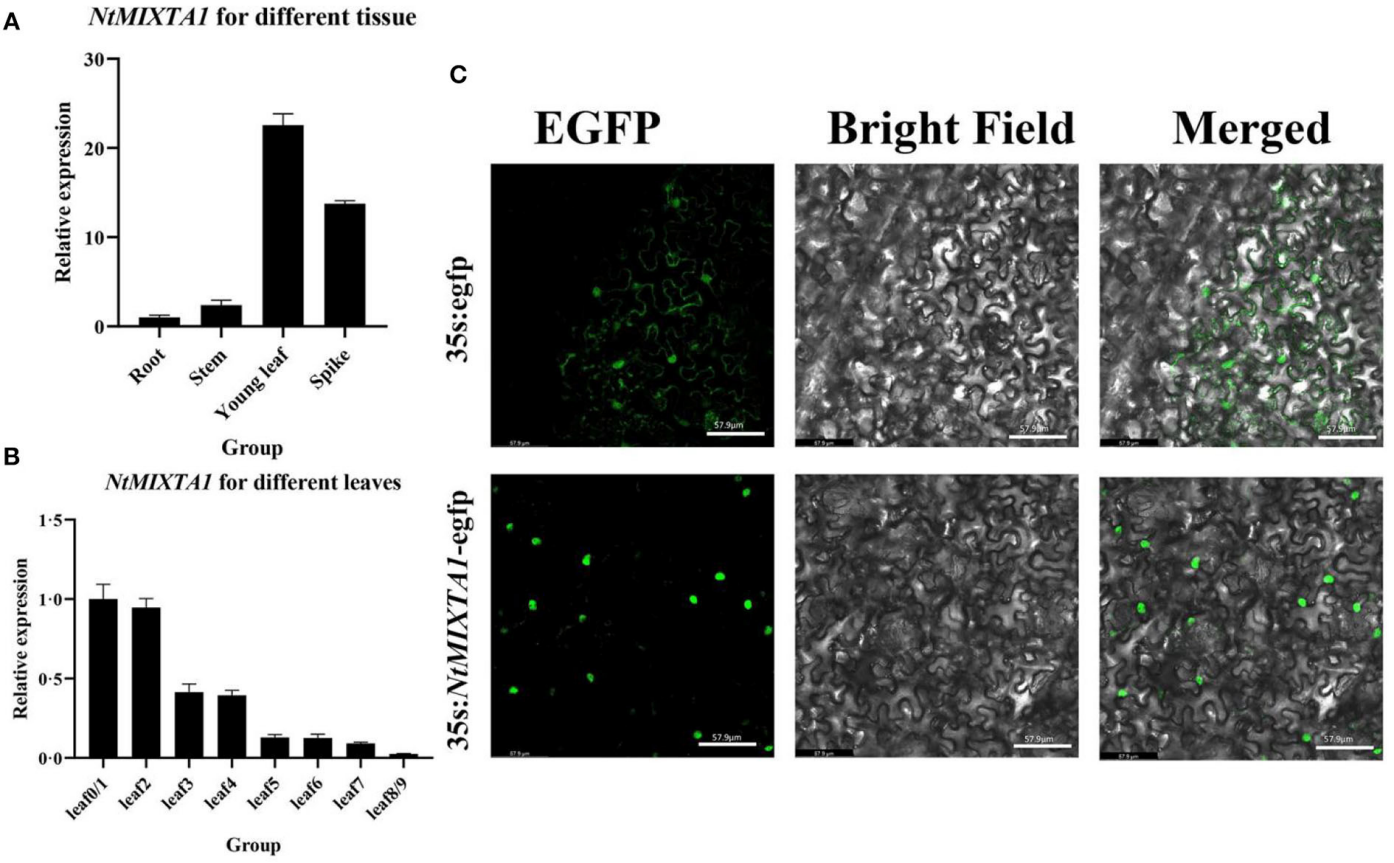
Relative expression of *NtMIXTA1* in different tissues **(A)** and leaves at different developmental stages **(B)** with β*-actin* as a reference gene. The relative expression levels were represented as mean ± SD (*n* = 3). **(C)** Subcellular localization of the StMIXTA protein in *Nicotiana benthamiana* epidermal cells. Bars = 57.9 μm.

The transcript levels of *NtMIXTA1* were investigated by RT-qPCR using cDNA from different tissues of root, stem, young leaf, and spike, and different levels of leaf 0–9. The results showed that *NtMIXTA1* exhibited higher expression levels in the young leaves and spikes, where PGTs are abundant. Among the different levels of leaves, *NtMIXTA1* was highly expressed in the youngest leaves (containing shoot leaf 0), and its expression decreased with leaf aging (Figures 2A,B); this was consistent with the PGT density variation (Jiang et al., 2016; Liu et al., 2021). The nuclear localization signal (NLS) of NtMIXTA1 was determined by sequence analysis. The subcellular localization of *NtMIXTA1* was investigated in *N. benthamiana* leaf cells. The GFP was fused to the N-terminus of NtMIXTA1, driven by the *35S* promoter. The GFP fluorescence of NtMIXTA1 was observed in the nucleus of *N. benthamiana* epidermal cells, while the fluorescence of the control was observed in whole cell (Figure 2C), suggesting that the NtMIXTA1 protein was located in the nucleus.

### *NtMIXTA1* overexpression in tobacco influences GT development

To explore the role of NtMIXTA1 in GT development, *NtMIXTA1* was overexpressed in a model plant, tobacco (*Nicotiana tabacum*). After genetic transformation, we identified 10 transgenic lines, named *NtMIXTA1*-OE1~10. The expression level of *NtMIXTA1* in these transgenic plants was higher than that in the wild-type (WT) tobacco, with the highest expression being ~40-fold higher than that in WT tobacco (Supplementary Figure S1A). We chose the lines OE8, OE9, and OE1 and collected their seeds (T1). Phenotypic analysis of leaves from T1 revealed that the *NtMIXTA1*-overexpressed tobacco plants showed a higher GT density and GT length than the WT plants which had a significant change between WT and OE lines with a *p-*value < 0.05 (Supplementary Figures S1B–E). In the *NtMIXTA1*-overexpressed lines, the density and length of GTs were increased around 1.59- and 1.65-fold compared with the control. These results also showed that NtMIXTA1 affected GT development and regulated GT length.

### *NtMIXTA1* silencing validates its involvement in PGT formation

**Figure 3:**
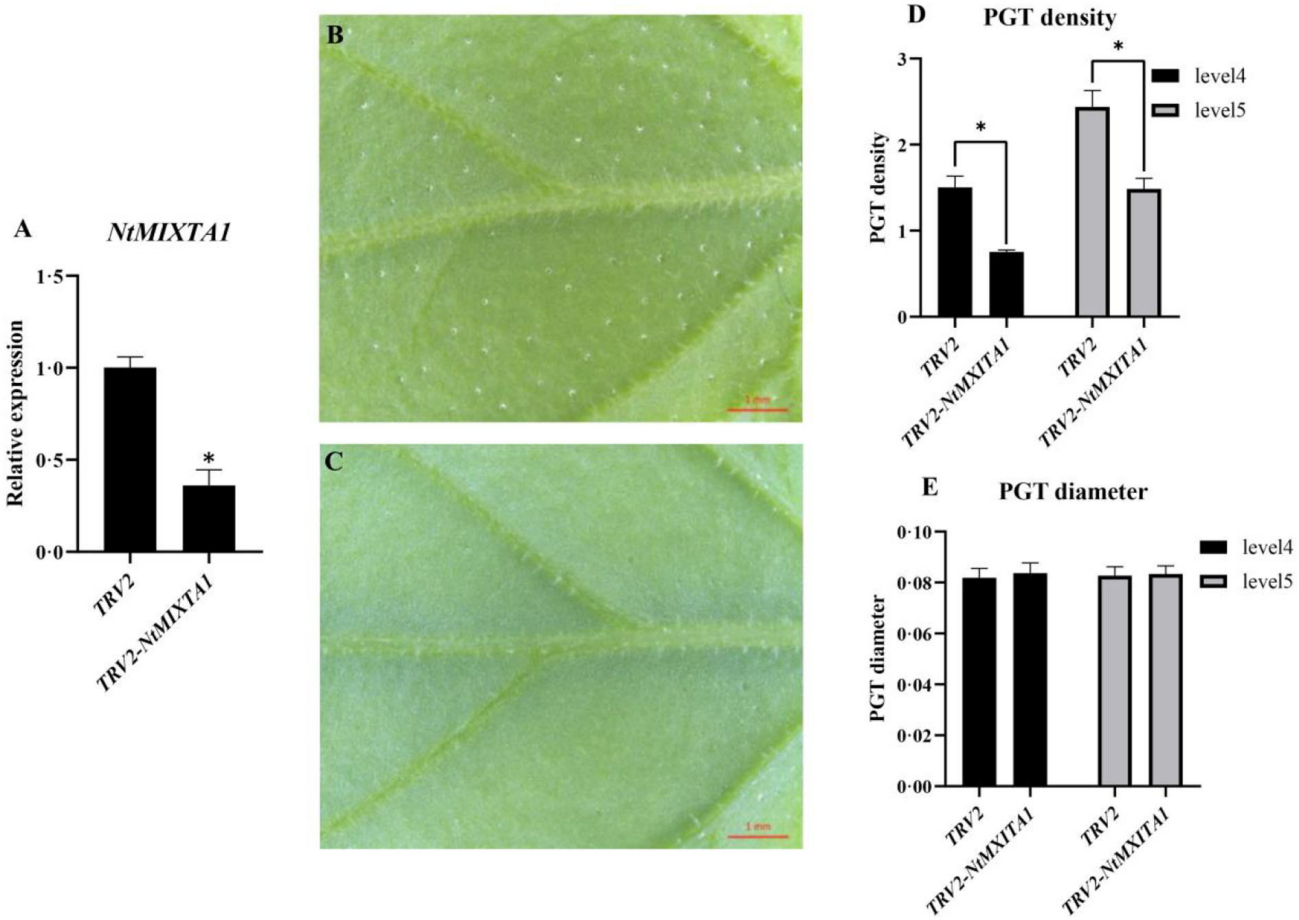
Gene expression and phenotype analysis of VIGS plants. **(A)** RT-qPCR analysis of *NtMIXTA1* expression with β*-actin* as a reference gene; the relative expression levels were represented as mean ± SD (*n* = 3); phenotypes of pTRV2 **(B)** and pTRV2-*NtMXITA1* **(C)** showed the presence and decline of PGTs; statistics of PGT density of pTRV2 and pTRV2-*NtMXITA1* **(D)** with *p-*value < 0.05; statistics of PGT diameter of pTRV2 and pTRV2-*NtMXITA1* **(E)**; phenotypes, the PGT diameter, and density were represented as mean ± SD (*n* = 5). ^*^was represented as p-value < 0.05.

The *NtMIXTA1* expression was silenced in *N. tenuifolia* seedlings by VIGS to determine its role in PGT initiation and development. Here, a marker gene, phytoene desaturase (*PDS*), was used to evaluate the gene-silencing efficacy by observing leaf photobleaching (Supplementary Figure S2). Based on the high *PDS*-silencing efficacy and albino leaf phenotype, the leaves of the fourth and fifth nodes were used as the experimental materials for VIGS. A fragment ~399 bp in size was PCR-amplified using primers specific for *NtMIXTA1* (Supplementary Table S1) as the target sequence, which was inserted into the VIGS vector pTRV2 to generate a new recombinant plasmid for gene silencing. The RT-qPCR analysis of *NtMIXTA1* showed that the expression of *NtMIXTA1* in pTRV*2-NtMIXTA1*-infected leaves was reduced by 64%, compared with that in pTRV2-infected leaves (Figure 3A) at 21 days post-infiltration, indicating that *NtMIXTA1* was effectively silenced in VIGS plants. *NtMIXTA1*-silenced *N. tenuifolia* exhibited a significant reduction in PGT density, whereas the morphology of the PGTs remained unchanged (Figures 3B–E). In detail, *NtMIXTA1*-silenced plants exhibited 49.94% reduction in leaf 4 and 39.00% reduction in leaf 5 in PGT density. These results suggested that the gene may play crucial role in regulating PGT formation.

### Monoterpene biosynthesis is linked to PGT formation

**Figure 4:**
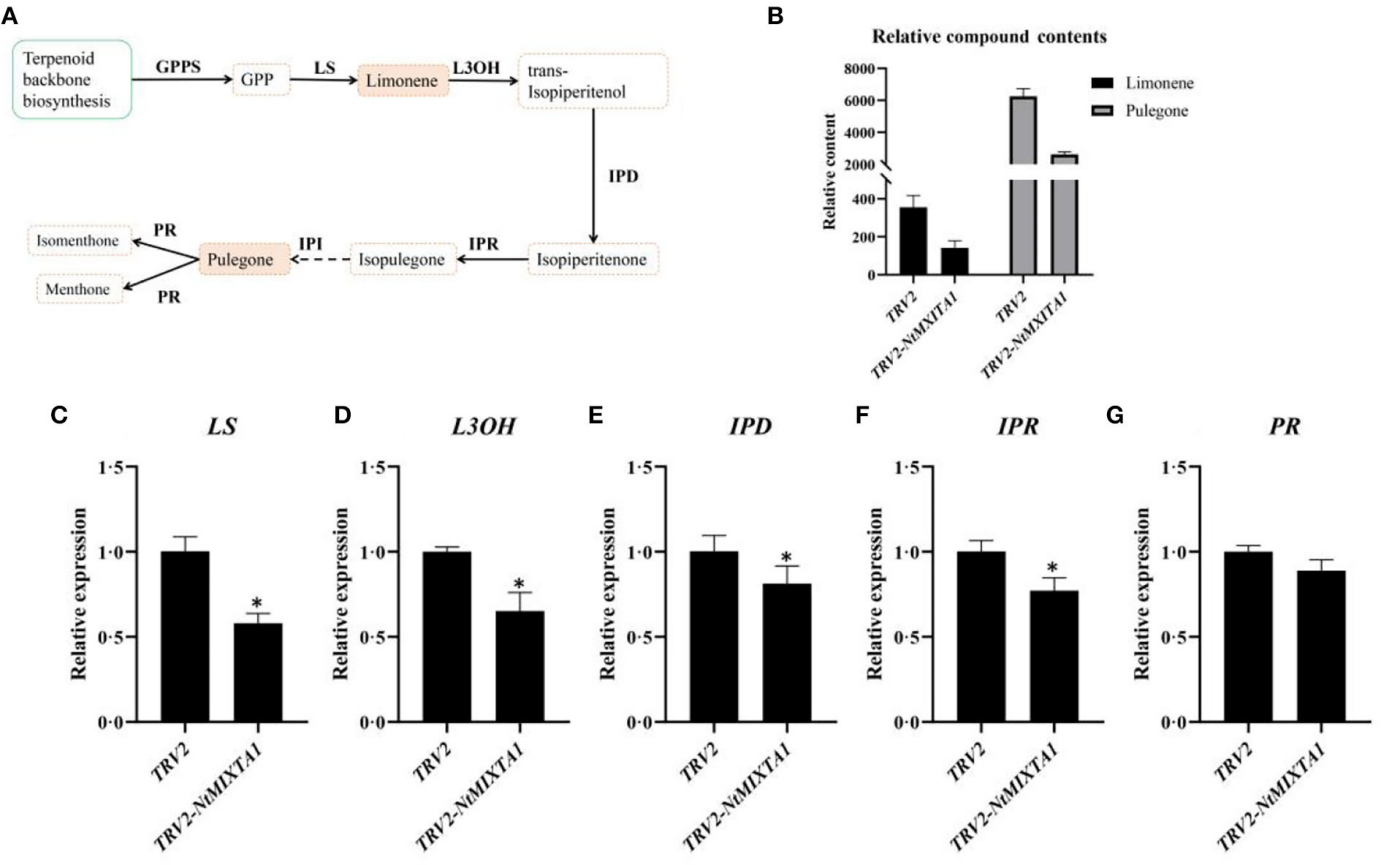
Variation of gene expression levels and compound contents of menthane monoterpenes biosynthesis. **(A)** The biosynthesis of menthane monoterpenes; **(B)** the relative contents of pulegone and limonene in TRV2 and in TRV2-*NtMIXTA1*; **(C–G)** the expression levels of *LS, L3OH, IPR, IPD*, and *PR* in TRV2 and in TRV2-*NtMIXTA1, p*-value < 0.05 with β*-actin* as a reference gene (*n* = 3); enzyme abbreviations: GPP, geranyl diphosphate; GPPS, geranyl diphosphate synthase; LS, limonene synthase; L3OH, limonene-3-hydroxylase; IPD, isopiperitenol dehydrogenase; IPR, isopiperitenone reductase; PR, pulegone reductase. ^*^was represented as p-value < 0.05.

PGTs are the predominant source of essential oils, especially menthane monoterpenoids, in *N. tenuifolia* (Liu et al., 2018). Monoterpene biosynthesis begins with the breakdown of geranyl diphosphate (GPP), which is catalyzed by limonene synthase (LS); this yields limonene, which then forms trans-isopiperitenol *via* a reaction catalyzed by L3OH (limonene-3-hydroxylase). Through the catalysis of isopiperitenol dehydrogenase (IPD) and isopiperitenone reductase (IPR), trans-isopiperitenol is converted into pulegone and then transformed into isomenthone or menthone by pulegone reductase (PR) (Figure 4A). Pulegone and limonene are the main ingredients in essential oils; thus, their contents are considered markers of essential oil content (Liu et al., 2018). Using GC analysis, we calculated the relative contents of these two compounds based on the peak areas with correction by referring to an internal standard. The peaks of pulegone and limonene were identified by using standard substances (Supplementary Figure S3). Compared with pTRV2-infected leaves, there was a significant reduction (>50%) in the relative levels of pulegone and limonene in the leaves of plants subjected to VIGS-mediated *NtMIXTA1* silencing (Figure 4B; Supplementary Figure S3; Supplementary Table S2). We inferred that fewer PGTs in *NtMIXTA1*-silenced plants reduced the levels of essential oils. Based on our previous study, the expression of key genes involved in menthane monoterpenoid biosynthesis may also be affected (Liu et al., 2018, 2021). Thus, we measured the expression levels of *LS, L3OH, IPR, IPD*, and *PR* by RT-qPCR. The expression levels of these genes in *NtMIXTA1*-silenced *N. tenuifolia* were substantially lower than those of pTRV2-treated plants (Figure 4C), which was consistent with our inference.

### Differential gene expression and differential metabolites in *NtMIXTA1*-silenced plants

To explore the regulatory networks that may underlie PGT formation and the related metabolic variation influenced by *NtMIXTA1*, transcriptomic and widely targeted metabolic analyses (LC-MS and GC-MS) were performed using leaves from TRV2 and VIGS-silenced TRV2-*NtMIXTA1* plants. The PCA analysis of RNA-seq results and metabolites showed good reproducibility for the samples whose biological replicates were closely grouped (Supplementary Figure S4).

For metabolic analysis, the VIGS samples were analyzed using widely targeted LC-MS (in both the negative and positive ion modes) and by GC-MS metabolic profiling. All the samples were mixed for quality control (QC) to confirm the repeatability and reliability of the method (Xiao et al., 2021). The total ion chromatograms (TICs) obtained after GC-MS and LC-MS showed that our data were reproducible and reliable (Supplementary Figures S5, S6). The relative contents of the corresponding compounds were calculated from each peak area. There were 1,705 identified metabolites, including 301 terpenoids, 201 flavonoids, 154 phenolic acids, 135 esters, 96 lipids, and others (Supplementary Table S2; Supplementary Figure S7). To identify the differentially expressed metabolites (DEMs) in the TRV2- and *NtMIXTA1*-silenced plants, metabolites were screened using the following criteria: variable importance in projection (VIP) value > 1 and *p*-value < 0.05 from the OPLS-DA model. Among these metabolites, 406 were downregulated and 183 were upregulated in *NtMIXTA1*-silenced plant, compared with the case in TRV2-silenced plant (Supplementary Table S2; Supplementary Figure S8). Terpenoids, flavonoids, and lipids accounted for 25%, 9%, and 8% of the total metabolites, respectively (Supplementary Figure S8B). These DEMs were mapped to 82 KEGG pathways (Supplementary Table S3).

For the construction of the transcriptome atlas, TRV2 and *NtMIXTA1*-silenced plants (three biological replicates) were analyzed; this yielded 40.98 Gb of clean data with high-quality reads (Q30 > 91%) without an adaptor and an average GC content > 48% for all libraries. After the genome alignment and *de novo* assembly of novel genes, we detected 20,930 genes, including 23,168 transcripts and 3,313 novel genes, with a fragments per kilobase of transcript per million fragments mapped (FPKM) value > 0 (Supplementary Table S4). In the TRV2 and *NtMIXTA1*-silenced plants, 1,523 differentially expressed genes (DEGs) were generated after screening, with 761 upregulated and 762 downregulated genes in *NtMIXTA1*-silenced leaves (Supplementary Table S4; Supplementary Figure S9). The DEGs were assigned to 122 KEGG pathways (Supplementary Table S3). We speculated that these DEGs and DEMs may be influenced by alterations in *NtMIXTA1* expression.

### *NtMIXTA1* silencing influences the biosynthesis of flavonoids, terpenoids, and lipids

**Figure 5:**
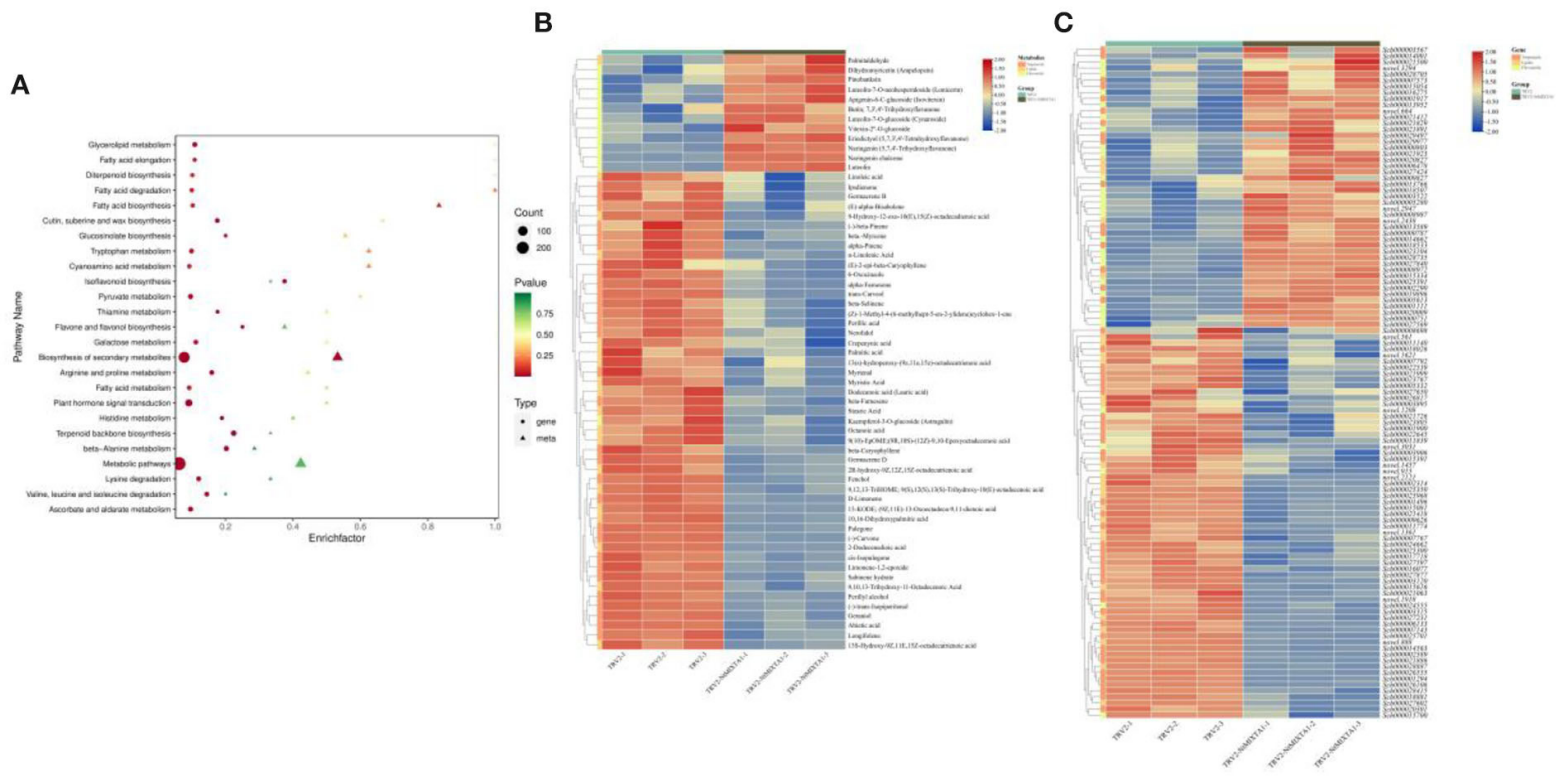
KEGG enrichment of top 50 pathways of combined analysis **(A)**. Heatmap of DEMs involving terpenoids, flavonoids, and lipids **(B)** and related gene expression **(C)**.

Transcriptome and metabolic profiling data were integrated to explore the influence of gene expression and metabolite variation on the occurrence of lower PGT density phenotypes resulting from *NtMIXTA1* silencing. Among the DEMs, 69% were downregulated in *NtMIXTA1*-silenced plants (Supplementary Figure S8A). The top three DEMs were terpenoids, flavonoids, and lipids (Supplementary Figure S8B), suggesting a close relationship between PGT formation and terpenoid, flavonoid, and lipid biosynthesis. In detail among the DEMs, 146 terpenoids, 55 flavonoids, and 49 lipids were identified (Figures 5A,B) (Supplementary Table S5). Cluster analysis showed that most of the terpenoids (118 downregulated) and lipids (32 downregulated) were detected at low levels in the *NtMIXTA1*-silenced plants (Figure 5C). Flavonoids comprised 26 downregulated and 29 upregulated metabolites (Supplementary Figure S10). The biosynthesis of flavonoids is known to include the following enriched KEGG pathways: biosynthesis of flavonoids (ko00941), flavones and flavonols (ko00944), anthocyanins (ko00942), and isoflavonoids (ko00943). Terpenoid-related biosynthesis includes the following enriched KEGG pathways: biosynthesis of terpenoid backbone (ko00900), monoterpenoids (ko00902), diterpenoids (ko00904), sesquiterpenoids, and triterpenoids (ko00909). Lipid-related biosynthesis includes the following enriched KEGG pathways: fatty acid biosynthesis (ko00061), fatty acid elongation (ko00062), fatty acid degradation (ko00071), cutin, suberin, and wax biosynthesis (ko00073), biosynthesis of unsaturated fatty acids (ko01040), fatty acid metabolism (ko01212), α-linolenic acid metabolism (ko00592), and linoleic acid metabolism (ko00591). Most of the biosynthesis pathways mentioned above were enriched in KEGG analysis of the transcriptome and metabolome data (Figure 5A). Therefore, we investigated the DEGs and DEMs involved in these biosynthetic pathways. In total, 54 DEGs and 30 DEMs were related to terpenoid biosynthesis, 30 genes and 12 metabolites were related to flavonoid biosynthesis, and 27 genes and 19 metabolites were related to lipid biosynthesis (Supplementary Table S5). Combined with the results of the KEGG-mapped DEGs and DEMs, we found that most terpenoid biosynthesis- and lipid biosynthesis-associated genes were notably downregulated in *NtMIXTA1*-silenced leaves; this finding was in accordance with the observed reduction in the contents of related metabolites (Figures 5B,C; Supplementary file 2).

**Figure 6:**
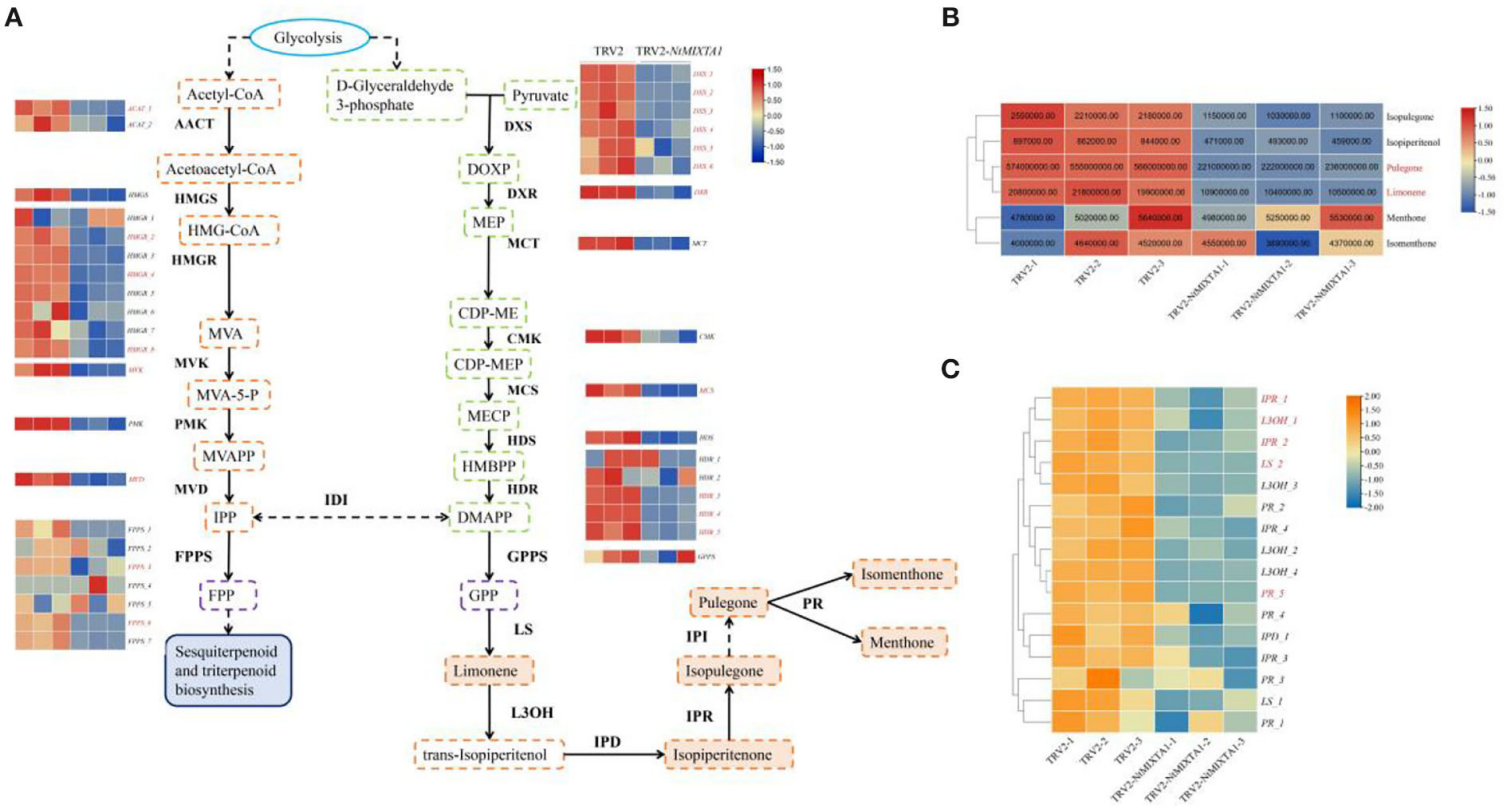
Biosynthesis of menthane monoterpene and variations involving related genes and metabolites; **(A)** biosynthetic pathway of menthane monoterpene biosynthesis; **(B)** heatmap of menthane monoterpenes content; **(C)** heatmap of gene expression in menthane monoterpene biosynthesis. Enzyme abbreviations: AACT, acetyl-CoA C-acetyltransferase; HMGS, hydroxymethylglutaryl-CoA synthase; HMGR, hydroxymethylglutaryl-CoA reductase; MVK, mevalonate kinase; PMK, phosphomevalonate kinase; MVD, diphosphomevalonate decarboxylase; DXS, 1-deoxy-D-xylulose-5-phosphate synthase; DXR, 1-deoxy-D-xylulose-5-phosphate reductoisomerase; MCT, 2-C-methyl-D-eryth-ritol 4-phosphate cytidylyltransferase; CMK, 4-diphosphocytidyl-2-C-methyl-D-erythritol kinase; MCS, 2-C-methyl-D-erythritol 2,4-cyclodiphosphate synthase; HDS, (E)-4-hydroxy-3-methylbut-2-enyl-diphosphate synthase; HDR, 4-hydroxy-3-methylbut-2-en-1-yl diphosphate reductase; IDI, isopentenyl-diphosphate Delta-isomerase; FPPS, farnesyl diphosphate synthase. GPPS, geranyl diphosphate synthase; LS, Limonene synthase; L3OH, limonene-3-hydroxylase; IPD, isopiperitenol dehydrogenase; IPR, isopiperitenone reductase; PR, pulegone reductase. The DEGs and DEMs are shown in red in the heatmap.

First, we focused on the biosynthesis of menthane monoterpenoids, which produced the main constituents of volatile oils in *N. tenuifolia* (Liu et al., 2018; Bai et al., 2021). Metabolites such as pulegone, limonene, and isopulegone were significantly downregulated in TRV2-*NtMIXTA1*, with similar trends in the expression of genes involved in the menthane monoterpenoid biosynthesis pathways (Figure 6). This finding is in accordance with the results of the GC analysis of the pulegone and limonene contents (Figure 4B) and RT-qPCR analysis of *LS, L3OH, IPR, IPD*, and *PR* expression levels (Figure 4C). The mevalonate (MVA) and 2-methyl-D-erythritol-4-phosphate (MEP) pathways generate the key precursors required for synthesizing several terpenoids: isopentenyl pyrophosphate (IPP) and dimethylallyl pyrophosphate (DMAPP) (Lange and Ahkami, 2013). As expected, the genes associated with these pathways, such as *DXS* and *DXR* (the core genes of MEP), and *HMGS* and *HMGR* (the core genes of MVA), were downregulated in the *NtMIXTA1*-silenced plants (Figure 6A). This may result in the reduced biosynthesis of IPP and DMAPP, leading to lower levels of terpenoids in *NtMIXTA1*-silenced plants (Figure 5B).

Second, trichome development may influence cuticle and wax biosynthesis (Panikashvili et al., 2007; Shi et al., 2018). Genes related to the above biosynthesis such as *CYP77A* (cytochrome P450 family 77 subfamily A) and *HHT1* (omega-hydroxypalmitate O-feruloyl transferase) were downregulated; the levels of cutin-related metabolites, such as hexadecanoic acid and octadecanoic acid, were reduced in *NtMIXTA1*-silenced plants (Supplementary file 2). Fatty acids are precursors of cuticle and wax (Panikashvili et al., 2007), and as a result, the reduction of the fatty acid contents in *NtMIXTA1*-silenced plants may influence cuticle and wax biosynthesis. In addition to the above genes, we also identified genes homologous to cutin and wax biosynthesis-related genes, such as *LAS2* (long-chain acyl-CoA synthetase 2), *KCS12* (3-ketoacyl-CoA synthase 12), *CYP77A1* (cytochrome P450 77A1), *CER10* (3-oxo-5-alpha-steroid 4-dehydrogenase family protein), and *KCS10* (3-ketoacyl-CoA synthase 10) (Supplementary Figure S11B); these were mostly downregulated in *NtMIXTA1*-silenced plants. In conclusion, our results demonstrated that the repression of *NtMIXTA1* transcript levels suppressed cuticle biosynthesis in *N. tenuifolia*.

Next, trichome formation is also associated with flavonoid biosynthesis (Kang et al., 2014; Li et al., 2016). According to our metabolic data, flavonoids can be classified into flavones, flavonols, flavanones, flavanonols, flavonoid carbonosides, isoflavones, and chalcones. There was no specific conversion of the different flavonoid structures (Supplementary Figure S12). Interestingly, the metabolites detected in flavonoid biosynthesis (ko00941), including naringenin chalcone, naringenin, eriodictyol, luteolin, and dihydromyricetin (Supplementary Figures S12A,B), were upregulated in *NtMIXTA1*-silenced plants. The genes encoding the enzymes that catalyze the formation of the compounds mentioned above, including *C4H* (cinnamic acid 4-hydroxylase), *F3H* (flavonoid 3-hydroxylase), and *F3'H* (flavonoid 3'-hydroxylase) (Supplementary Figure S12C), were also upregulated in *NtMIXTA1*-silenced plants. Understanding the relationship between flavonoid flux and PGT formation may require further research on the regulatory networks associated with these processes.

## Discussion

### Analysis of gene function using VIGS

VIGS is a useful tool for investigating gene functions in species where stable genetic transformation is difficult or impossible to achieve (Baulcombe, 1999; Lange et al., 2013; Fei et al., 2021). This method requires less energy and time to execute because it achieves transient transformation (Lu et al., 2003). In addition, VIGS can efficiently identify genes that cannot be examined using other techniques. The method has been widely used in diverse areas of plant research, including evolutionary developmental biology, analyses of secondary metabolism, symbiotic interactions, and plant–pathogen interactions (Dommes et al., 2019). Recently, TRV has been used to determine the functions of genes associated with GT formation and development (Janga et al., 2019; Zang et al., 2021). TRV1 and TRV2 are one set of the most widely used vectors (Ratcliff et al., 2001) that have been used to silence genes in *Nicotiana benthamiana*, tomato, petunia, *Arabidopsis*, cotton, and cucumber (Liu et al., 2004; Fu et al., 2005; Fang et al., 2021). The *PDS* gene is a well-known reporter used in the VIGS system, and *PDS*-silenced plants exhibit the photobleaching phenotypes (Liu and Page, 2008). Both the *GoSPGF* in cotton, and the *CsHOX3* and *CsbHLH1* in *Cucumis sativus* L have been shown to play a role in trichome formation (Janga et al., 2019; Dong et al., 2022).

In this study, we established a VIGS system in *N. tenuifolia* using *PDS* as a control for VIGS efficiency. *PDS* silencing persisted for ~40 days and its silencing efficiency was the highest in leaves of the fourth and fifth nodes (from root to shoot), with an obvious albino leaf phenotype (Supplementary Figure S2). These leaves were therefore collected as the plant material for analysis. The silencing effect of VIGS decreased with plant growth. RT-qPCR results demonstrated that *PDS* expression was silenced until the 9th leaf pair (Supplementary Figure S2). There was no difference in leaf phenotypes between *PDS*-silenced and TRV2-infected plants. In addition, for the specificity of VIGS silencing *NtMXITA1*, the similarity of the nucleic acid sequence (399 bp) between VIGS fragments and other genes was examined. The VIGS fragments exhibited a similarity of 69, 40, and 8% to Sch000016802, Sch000000071, and Sch000018969, respectively. Gene count and RT-qPCR revealed that the transcript levels of these three additional genes did not differ between TRV2 and TRV2-NtMXITA1 lines (Supplementary Figure S13). These results indicated that *NtMIXTA1* VIGS had no off-target effects. As a result, based on the VIGS system, *NtMIXTA1* was silenced, leading to a decrease in PGT density and terpenoids levels. Then, RNA-seq and metabolic analysis of *NtMIXTA1*-silenced plants were performed to investigate regulatory networks involved in GT development and GT development-associated genes and metabolites.

### *NtMIXTA1* positively influences PGT formation and monoterpene biosynthesis

R2R3 MYB SBG9 genes regulate the differentiation of epidermal projections into trichomes and conical cells, including petal epidermis regulation of AmMIXTA, trichome development regulation of *AmMYBML1, AmMYBML2, AtMYB106, AtMYB16*, and Aa*MIXTA1* (Perez-Rodriguez et al., 2005; Baumann et al., 2007; Jakoby et al., 2008; Shi et al., 2018). In this study, 32 candidate genes for the R2R3 MYB SBG9 were identified. According to the ML phylogenetic tree and protein alignment, the gene *NtMIXTA1* was identified and further research was conducted (Figure 1A). We attempted to silence *NtMIXTA1* in *N. tenuifolia* seedlings using VIGS, which resulted in a decreased density of PGTs compared to the controls but did not affect PGTs morphology (Figures 3B–E).

AaHD1 and AaHD8 also promote GT initiation in *A. annua* (Yan et al., 2017; Xie et al., 2021). *HD1* and *HD8* genes in *N. tenuifolia* are homologous to *AaHD1* and *AaHD8*, and their expression was also reduced in *NtMIXTA*-silenced plants (Supplementary Figure S11A). AaHD8 interacts with AaMIXTA1 and promotes the expression of *AaHD1*, which regulates GT initiation and cuticle development, *via* the HD ZIP-MYB complex in *A. annua* (Yan et al., 2018). In tomatoes, a *SlMIXTA*-like gene also cooperates with HD-ZIP IV TFs to regulate cuticle biosynthesis and epidermal cell formation (Lashbrooke et al., 2015). The relationship between NtMIXTA1, HD1, and HD8 may also influence GT development, requiring additional research.

Previous research has demonstrated that menthane monoterpenoid biosynthesis in *N. tenuifolia* occurs mainly in the PGTs (Liu et al., 2021). Metabolic studies of *NtMIXTA1*-silenced plants demonstrated that the production of monoterpene biosynthesis was downregulated in *N. tenuifolia* (Figures 4, 6B,C). RNA-seq analysis was also used to evaluate the expression levels of genes involved in menthane monoterpenoid biosynthesis, including *LS, L3OH, IPD, IPR*, and *PR*; these findings were consistent with the RT-qPCR results showing downregulated (Figures 4C, 6C). In addition, the gene transcription of MVA and MEP, which act upstream of terpenoid biosynthesis, was repressed in *NtMIXTA1*-silenced plants resulting in low terpenoid levels (Figure 6A). Based on the above variation, we inferred that *NtMIXTA1* may affect PGT initiation and the expression of metabolic genes.

### A complex network of PGT formation, terpenoids, flavonoids, and lipids

Fatty acids are the primary components of lipids such as cutin and wax. Previously research has shown that the morphology of the cuticle and trichome (both GTs and NGTs) is altered in both trichome and cuticle mutants, implying that the trichome and cuticle are involved in such an interaction (Berhin et al., 2021). For example, the function loss of AtGL1 and Atmyb106 showed a reduction in cutin load and abnormal trichomes (Xia et al., 2010; Oshima et al., 2013); SlMX1, SlWOOLLY, and AaMIXTA all regulate wax biosynthesis and GT initiation (Mohamed et al., 2016; Chang et al., 2018; Shi et al., 2018; Xiong et al., 2020). These findings imply an interaction between trichome and cuticle development. In this study, a similar trend was observed with PGT development and cutin deposition. The expression of genes related to cutin and wax biosynthesis, including *LAS2, KCS12, CYP77A1, CER10*, and *KCS10*, as detected by RNA-seq analysis, and cutin-related metabolites, including fatty acids, was reduced in *NtMIXTA1*-silenced plants (Figure 5C; Supplementary Figure S11B; Supplementary file 2).

In addition to the cuticle and trichome, the impact of flavonoid and terpenoid biosynthetic pathways, and trichome development was recently demonstrated in tomato and *A. annua* (Kang et al., 2014; Shi et al., 2018; Sugimoto et al., 2021). The *af* mutated tomato, *AaYABBY5-*overexpressed, and *AaTAR2-*overexpressed *A. annua* all affect trichome density, flavonoid, and terpenoid biosynthesis (Kayani et al., 2019, 2021; Zhou et al., 2020; Sugimoto et al., 2021). Our findings demonstrated that terpenoid-related genes and terpenoid metabolites were downregulated in plants with fewer PGTs, compared to controls; however, the variation in flavonoid contents in control plants and plants with fewer PGTs was not significant. The reason may be that the VIGS method involved transient transformation, which resulted in temporary gene silencing. Although the expression of genes involved in flavonoid biosynthesis was upregulated, protein translation may not have been affected at the sampling time. Flavonoids and terpenoids are specifically synthesized and accumulated in the tVI-GTs (type VI GTs) of tomatoes; however, flavonoids have not been detected in the PGTs of *N. tenuifolia*. The aforementioned reasons may lead to differentiation in metabolites levels between our study and previous reports. Our metabolic analysis detected 201 flavonoids in total, 79 of which were flavonoid glycosides generated by glycosyltransferases (UFGTs); therefore, the levels of UFGTs would also be influenced. Any correlation between the metabolic pathways of flavonoids and terpenoids in future research would be valuable.

**Figure 7:**
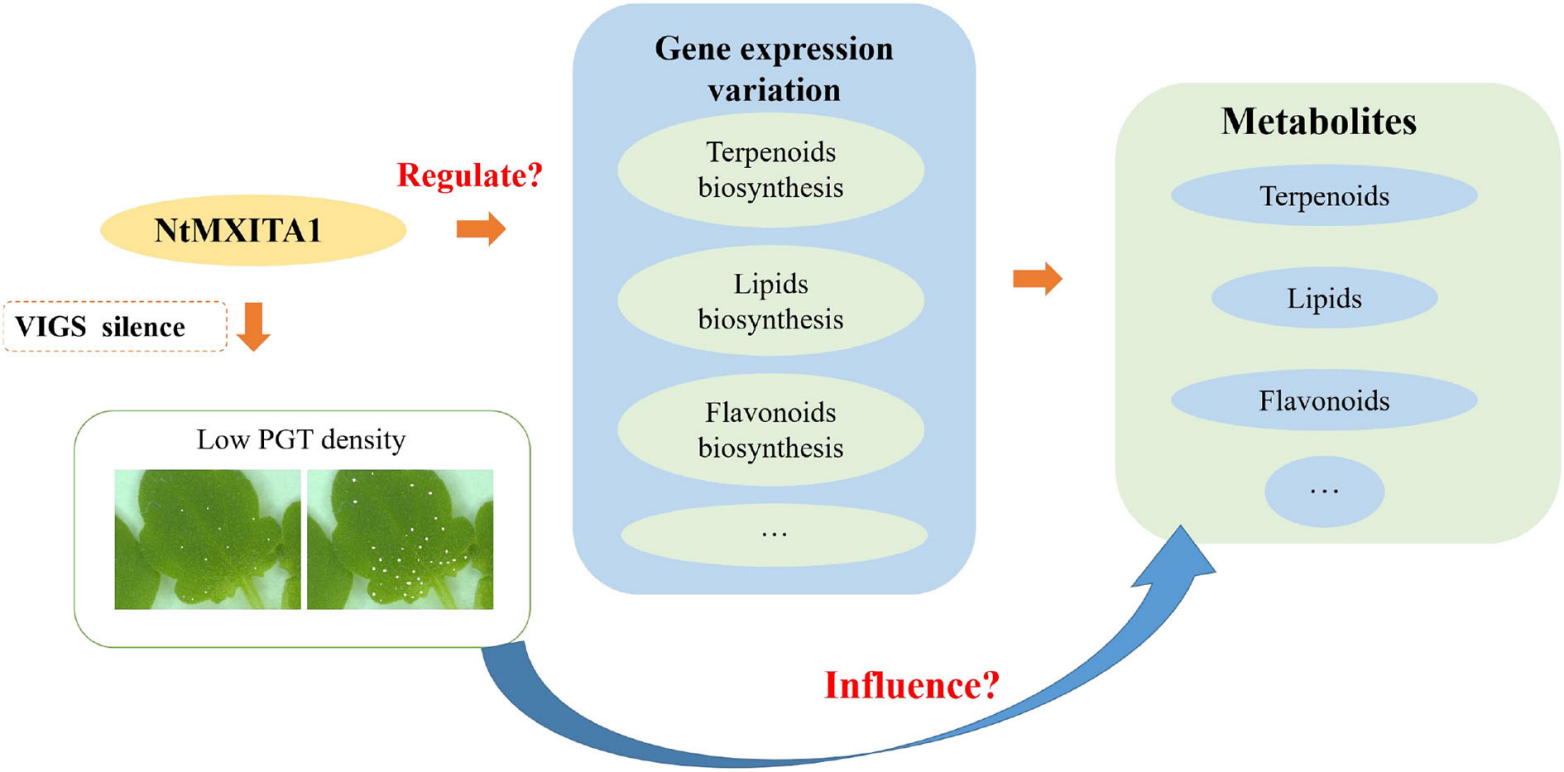
Hypothesis model of *N. tenuifolia* PGT initiation and metabolite variation.

There were 146 DEGs were annotated as TFs (77 downregulated and 69 upregulated). The top four downregulated TF families were AUX/IAA, MYB, WRKY, and AP2/ERF-ERF. The MYB and WRKY were essential in regulating plant development and secondary metabolism (Ma and Constabel, 2019; Chalvin et al., 2020; Xie et al., 2020; Fu et al., 2021). The AP2/ERF was an important regulator in plant morphogenesis, stress response mechanisms, hormone signal transduction, and metabolite regulation (Feng K. et al., 2020). The AUX/IAA typically affected plant growth and secondary metabolism *via* endogenous hormones (Salehin et al., 2019; Wei et al., 2021). Therefore, the other possibility is that NtMIXTA1 can combine with other TFs to regulate the above biosynthesis and PGT initiation. The following hypothesis model is based on the above results (Figure 7). Silencing of the *NtMIXTA1* resulted in lower PGT density, gene expression variation of terpenoids, lipids, flavonoids, and other biosynthesis, which finally resulted in the changes in the levels of terpenoids, lipids, and flavonoids. The regulated network will be investigated in subsequent studies.

## Conclusion

In summary, in this study, the R2R3 MYB SBG9 transcription factor *NtMIXTA1* was identified and characterized. The function of *NtMIXTA1* in GT formation and development was investigated by VIGS and transgenic tobacco. The results suggested NtMIXTA1 positively influenced PGT formation, and changes in the levels of terpenoids, lipids, and flavonoids. The VIGS system was established for *N. tenuifolia* to explore gene function rapidly. This study provides valuable information regarding the relationship between GT initiation and metabolic flux.

## Materials and methods

### Plant materials

The *N. tenuifolia* plants were grown at the Nanjing University of Chinese Medicine; the seeds were obtained from Hebei Province, China (Liu et al., 2018). The plants were grown in a greenhouse under 10,000 lux intensity and 50% humidity with a light:dark photoperiod of 16:8 h at 25°C. Plant tissues were carefully removed, immediately snap-frozen in liquid nitrogen, and stored at −80°C for RNA extraction. *Nicotiana benthamiana* (K326) seeds were grown in MS (Murashige and Skoog) medium and used in transgene experiments.

### Gene expression analysis, phylogenetic tree construction, and amino acid sequence alignment

Genes from the R2R3 MYB SBG9 are known to be the main regulators of epidermal cell development in various tissues, such as in the trichomes (Brockington et al., 2013). Genes belonging to the SBG9 R2R3 MYB were identified from transcriptome data. The gene expression levels were represented by count in RNA-Seq. A phylogenetic tree was constructed using the maximum-likelihood (ML) method using MEGA X software with 1,000 bootstrap-based JTT + G + I amino acid substitution models (Nei and Kumar, 2000; Kumar et al., 2018). Protein sequences of SBG9 R2R3 MYB genes from other species whose functions in epidermal differentiation are clear were downloaded from the National Center for Biotechnology Information (NCBI). Multiple amino acid sequence alignments of *NtMIXTA1* and the other proteins were performed using DNAMAN. The PCR primers used to amplify the *NtMIXTA1* ORF are listed in Supplementary Table S1.

### Subcellular localization of *NtMIXTA1*

The ORFs of *NtMIXTA1* were amplified by PCR, and this ORF was cloned into the pNC-cam1304-subN vector with the GFP protein-coding sequence (Yan et al., 2019) using the ClonExpress recombination reaction (Vazyme Biotech, Nanjing, China Nanjing, China Nanjing, China). The recombinant plasmid was then transformed into A*grobacterium tumefaciens* strain GV3101 and injected into *N. benthamiana* leaves (Sparkes et al., 2006). After administration, GFP signals were observed using a Leica TCS SP8 microscope under excitation and emission wavelengths of 488 and 507 nm, respectively (Leica Microsystems, Wetzlar, Germany); three biological replicates were analyzed for each sample.

### Over-expression vector construction and the transformation of WT and transgenic tobacco plants

The *NtMIXTA1* ORF was inserted into the pNC-Cam1304-35S to generate a recombinant overexpression plasmid. The correctness of the recombinant constructs was verified using colony PCR and DNA sequencing. A positive colony was used to extract the correct recombinant plasmid, which was then introduced into EHA105 cells. The overexpression vector transformed into tobacco was used, as described in a previous study (Ma et al., 2019).

RNA was extracted from the transgenic plants, and the universal primer pNC-Cam1304-35S (F: agcggataacaatttcacacagga; R: cgccagggttttcccagtcacgac) was used to select the positive transgenic plants. The expression of *NtMIXTA1* in the transgenic plants was quantified using RT-qPCR; the top three plants were grown in a greenhouse, and transgenic seeds (T1) from these plants were collected. Then, the density and length of the GTs from the transgenic plants T1 were observed and compared with those of the GTs from the WT plants.

### VIGS assay

A 399-bp fragment of *NtMIXTA1* was amplified by PCR from cDNA using a 2 × Phanta^®^ Max Master Mix (Vazyme Biotech, Nanjing, China Nanjing, China Nanjing, China). The primers used are listed in Supplementary Table S1. The target genes were inserted into pTRV2, which is referred to as pTRV2-*NtMIXTA1*. VIGS was performed as described in a previous study (Zang et al., 2021). In our experiment, cotyledons of 10-day-old seedlings of *N. tenuifolia* were injected. Empty pTRV1 and pTRV2 vectors were injected to represent the controls. The phytoene desaturase *(PDS)* gene was used as a marker for the silencing effect (Yamamoto et al., 2021). The leaves were collected for analysis approximately 21 days after injection.

### Microscopy and RT-qPCR analysis

After VIGS administration, leaves of the fourth and fifth nodes (counted from root to shoot) were used to observe PGT density, number, and morphology (Liu et al., 2018). The diameters of the PGTs were also recorded. These experiments were performed using five sample replicates.

The biological replicates were three times selected randomly from five VIGS samples. RNA isolation and cDNA generation for RT-qPCR were performed as described previously (Zhou et al., 2021). RT-qPCR was performed on a QuantStudio 3 Real-Time PCR System (Thermo Fisher Scientific Inc., Waltham, MA) using the ChamQ Universal SYBR qPCR kit (Vazyme Biotech). The RT-qPCR analysis and calculation of the relative gene expression levels were performed as described in a previous study (Zhou et al., 2021). All the primers used for the RT-qPCR analyses are listed in Supplementary Table S1.

### Measurement of the levels of essential oils

Leaves from plants subjected to VIGS were harvested for microscopic analysis. Each sample (0.1 g) was accurately weighed and extracted using n-hexane and camphor as an internal standard (final concentration 30 ng/μL). The mixture was ground and treated with ultrasound three times (60 Hz, 30 s). The extracts were collected in bottles after dehydration. The supernatant was collected after centrifuging the mixture and stored at −80°C. The contents of the volatile components were measured using a GC-FID instrument (7890A, Agilent Technologies, California) using an HP-5 column (30 m × 320 μm × 0.25 μm; Agilent 19091 J-413) with helium as a carrier gas. The detailed conditions for GC-MS and metabolite identification have been described in a previous study (Liu et al., 2018). The peak areas of the compounds were corrected using an internal standard, and the relative contents of each substance were calculated. All the experiments were performed in triplicate.

### RNA-seq of the VIGS plants

Total RNA was isolated from the leaves of the plants subjected to VIGS, with three biological replicates sampled for each tissue. Quality control for RNA and RNA-seq library construction was performed as described in a previous study (Liu et al., 2021). After the quality control of the raw data, a total of 40.98 Gb of clean data were generated. The FPKM values were noted for ascertaining gene expression, and DEGs were analyzed using featureCounts v1.6.2 and DESeq2 v1.22.1. A *p*-value < 0.05 and |log2foldchange| ≥ 0.5 (pTRV2-NtMIXTA1 vs. pTRV2) were used as thresholds for significant difference in expression. Gene functions were annotated using the GO, KEGG, Swiss-Prot, and NCBI-NR (non-redundant) databases.

### Metabolite extraction and analysis

Sample extraction of the widely targeted metabolome for LC-MS and GC-MS analyses has been described previously (Chen et al., 2013; Yuan et al., 2022). The detailed methods are listed in Supplementary file 1.

The volatile compounds were identified based on their spectra and retention times (RTs) using commercially available standards or characteristic fragment ions in an independent database constructed based on standard compounds. Specific ion-detection modes were used for accurate scanning, including the scanning typical RTs and qualitative and quantitative ions. As previously described, one quantitative ion and two–three qualitative ions were selected for each compound (Yuan et al., 2022). Quantitative ions were selected to integrate and correct the peak areas for further data processing. The metabolites detected by LC-MS were identified based on a standard compounds database, per standard metabolic operating procedures. Multiple-reaction monitoring (MRM) was used to measure the levels of the metabolites. Correction and calculation of the peak areas of the metabolites were performed as described in a previous study (Fraga et al., 2010). All the samples were analyzed in triplicate. Unsupervised principal component analysis (PCA), hierarchical cluster analysis (HCA), and orthogonal partial least squares discriminant analysis (OPLS-DA) of the identified metabolites were performed using R software. The metabolites were identified as differentially expressed metabolites when the following criteria were fulfilled: variable importance in projection (VIP) value ≥ 1 and *p-*value < 0.5 (Zeng et al., 2020).

## Data availability statement

The datasets presented in this study can be found in online repositories. The names of the repository/repositories and accession number(s) can be found at: https://www.ncbi.nlm.nih.gov/, PRJNA813002.

## Author contributions

PZ, CL, and QW designed the experiments and wrote the manuscript. PZ performed experiments with the help from JD, SD, WY, and ZS. PZ, JD, and ZS analyzed the data. All authors read and approved the final manuscript.

## Funding

This work was supported by the Natural Science Foundation of China (81903756 for CL, 81973435 and 81473313 for QW), 2017 Chinese Medicine Public Health Service Subsidy Project National General Survey of Traditional Chinese Medicine Resources [Finance Society (2017) No. 66], and 2018 Study on Variety research and quality characteristics of Dao-di herbs produced in Jiangsu Province for QW and Ecological Planting and Quality Assurance Project of Authentic Medicinal Materials (2021) for QW. The Postgraduate Research & Practice Innovation Program of Jiangsu Province (KYCX21_1759 and KYCX22_2031) for PZ.

## Conflict of interest

The authors declare that the research was conducted in the absence of any commercial or financial relationships that could be construed as a potential conflict of interest.

## Publisher's note

All claims expressed in this article are solely those of the authors and do not necessarily represent those of their affiliated organizations, or those of the publisher, the editors and the reviewers. Any product that may be evaluated in this article, or claim that may be made by its manufacturer, is not guaranteed or endorsed by the publisher.
