# Supplementary material for: Identification and characterization of a novel gene involved in glandular trichome development in Nepeta tenuifolia
Source: Front Plant Sci. 2022 Jul 29;13:936244. doi: 10.3389/fpls.2022.936244 (PMC9372485; doi:10.3389/fpls.2022.936244)
Supplement: Supplementary file 8 [file Data_Sheet_1.PDF]

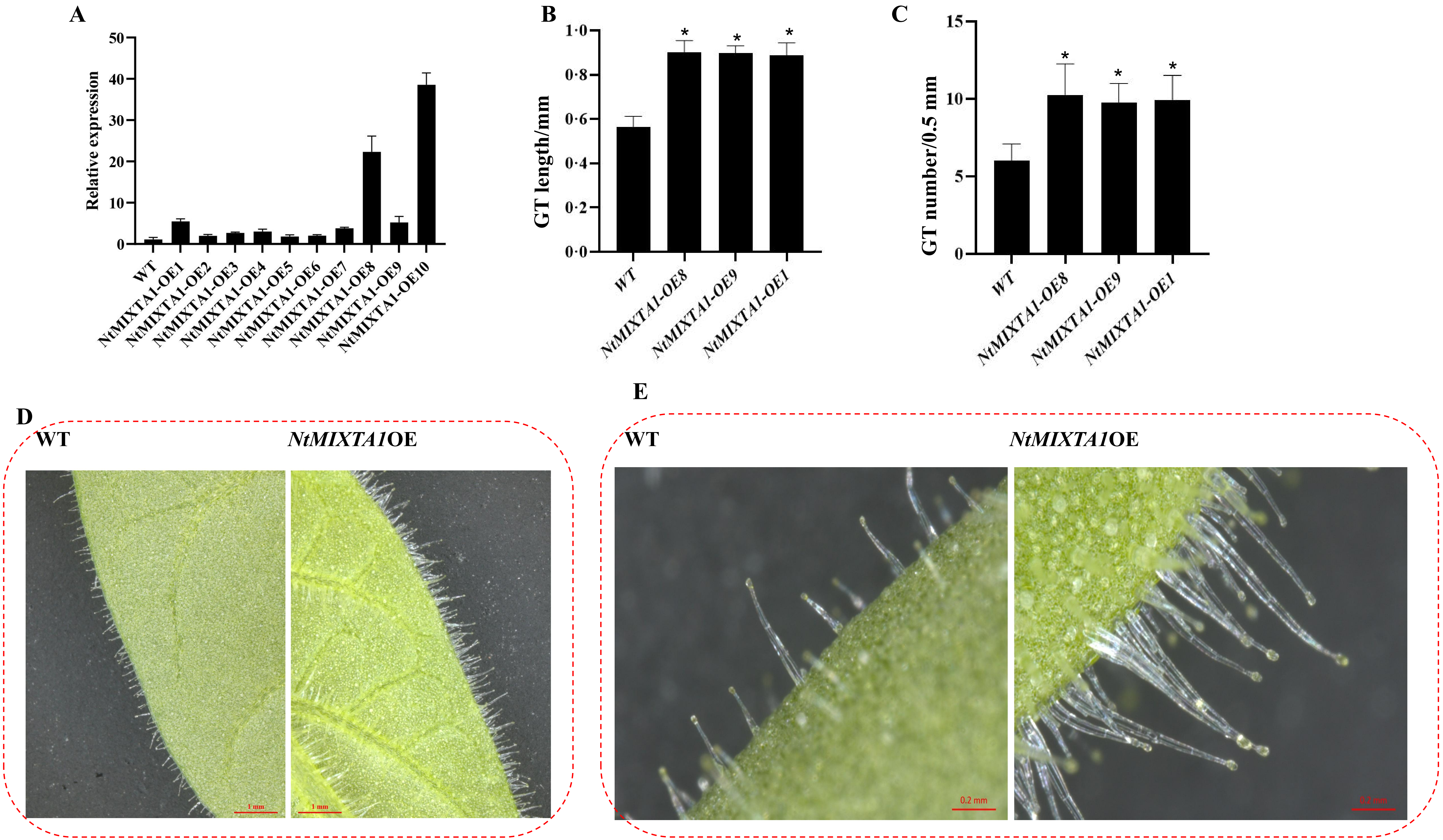

Figure S1. The NtMIXTA1 expression in transgenic lines of tobacco (A). The GT length (B) and number per 0.5 mm (C). The image of WT and transgenic tobacco (D-E).

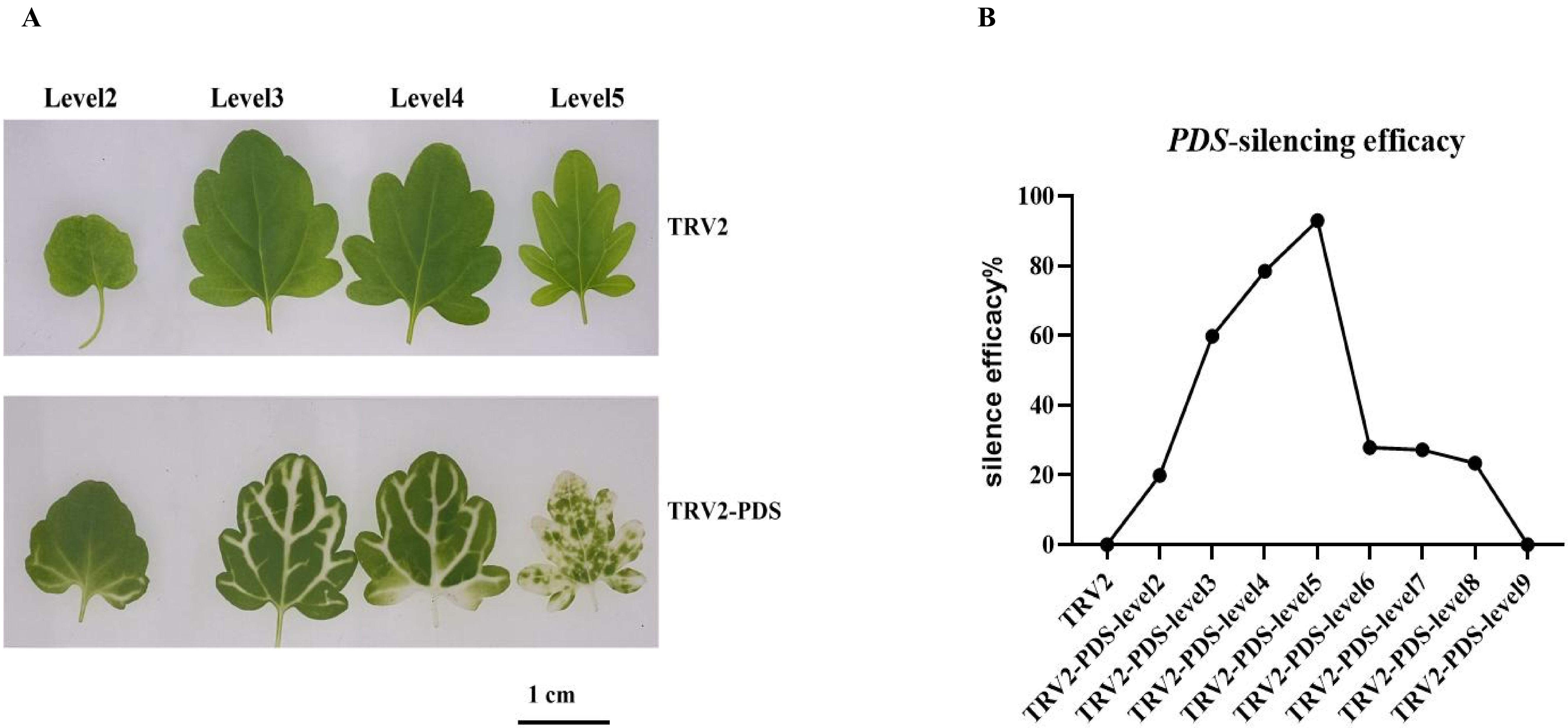

Figure S2. The bleaching phenotype resulting from silencing of PDS about different leaves (A) and PDS-silencing efficacy of leaf 2 to leaf 9 (B).

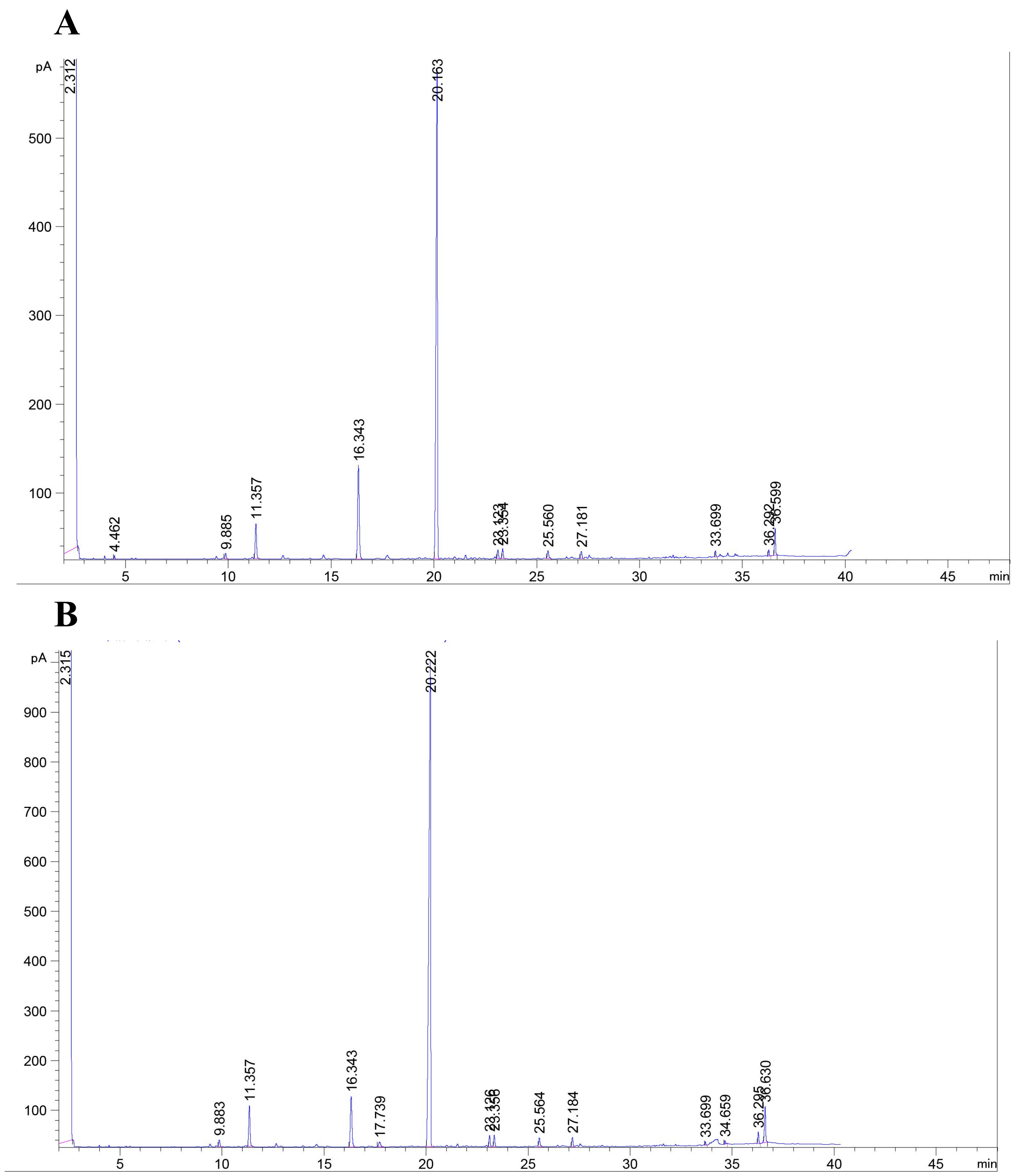

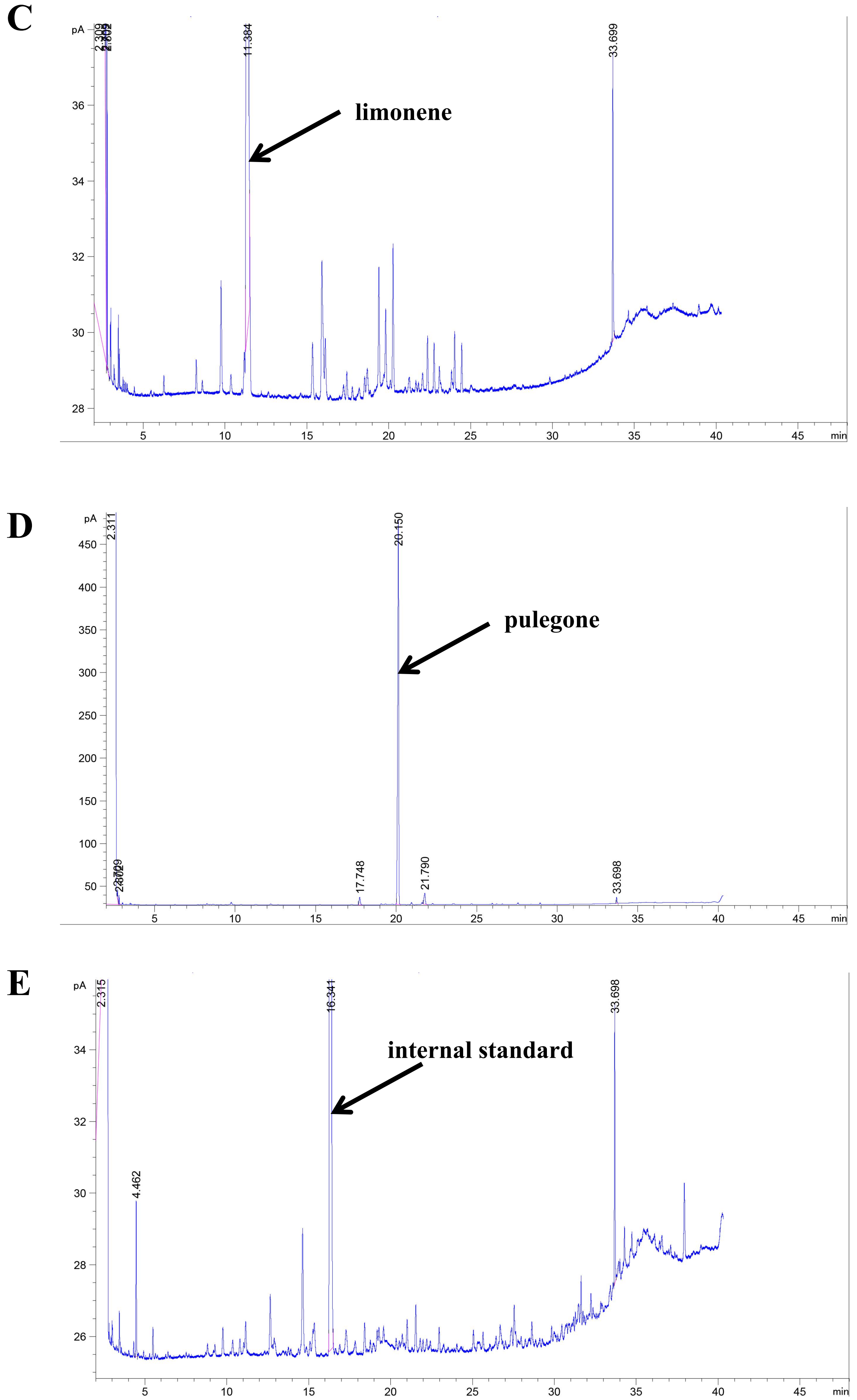

Figure S3. The GC chromatogram of TRV2-*NtMIXTA1* (A), TRV2 (B), limonene standrad (C), pulegone standrad (D), and internal standard (E).

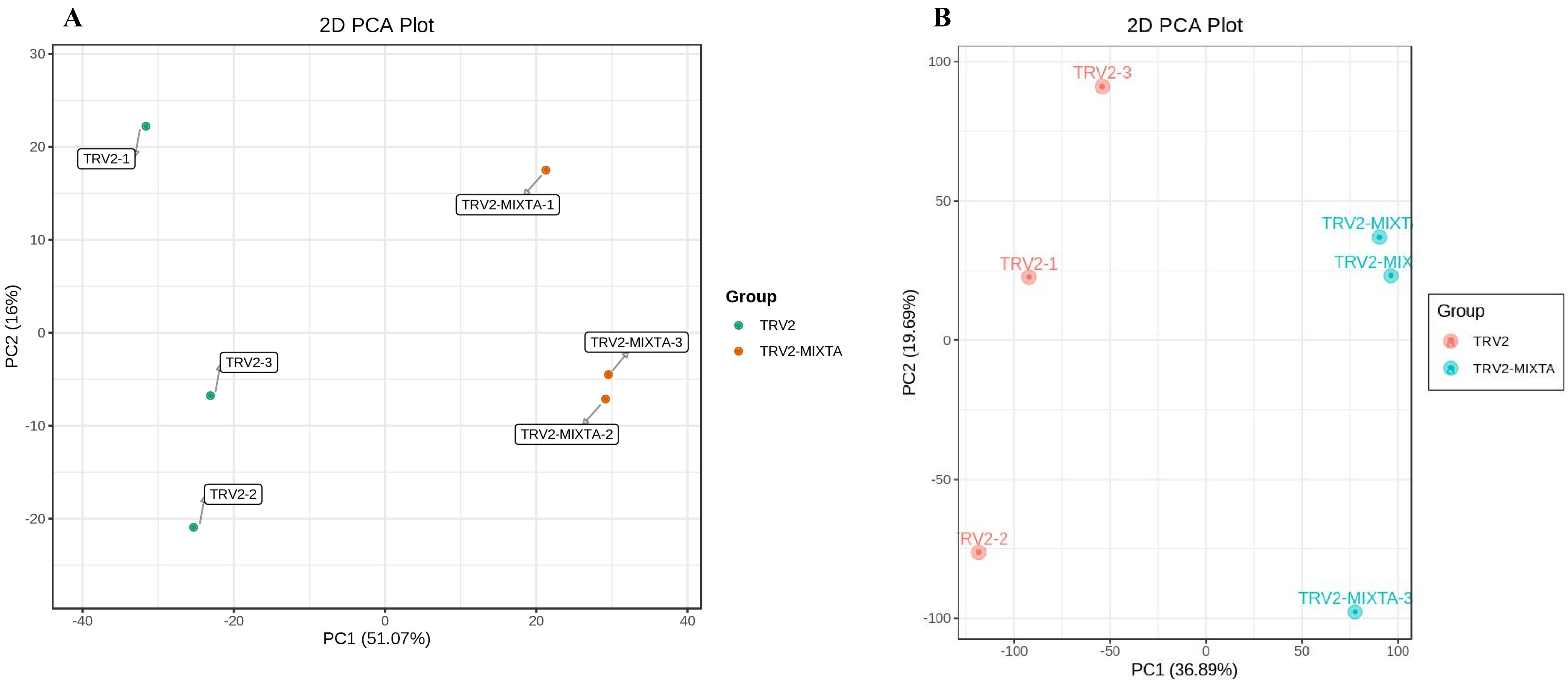

Figure S4. PCA analysis of metabolites and genes identified from TRV2 and TRV2-*NtMIXTA1*.

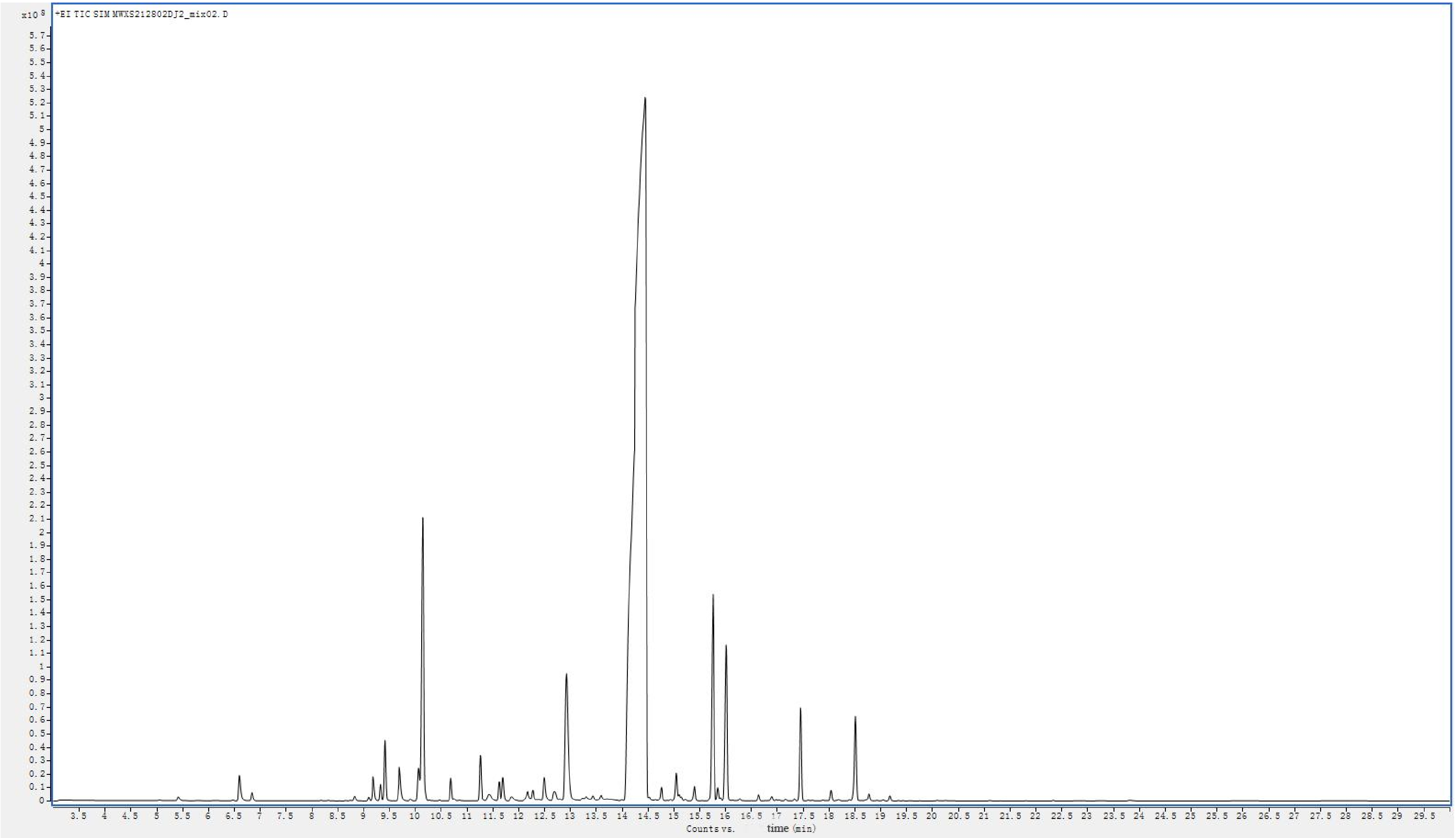

Figure S5. The TIC of GC-MS of mixed sample. The abscissa represents the retention time (min) of metabolite detection, and the ordinate represents the intensity of the ion current (cps: count per second).

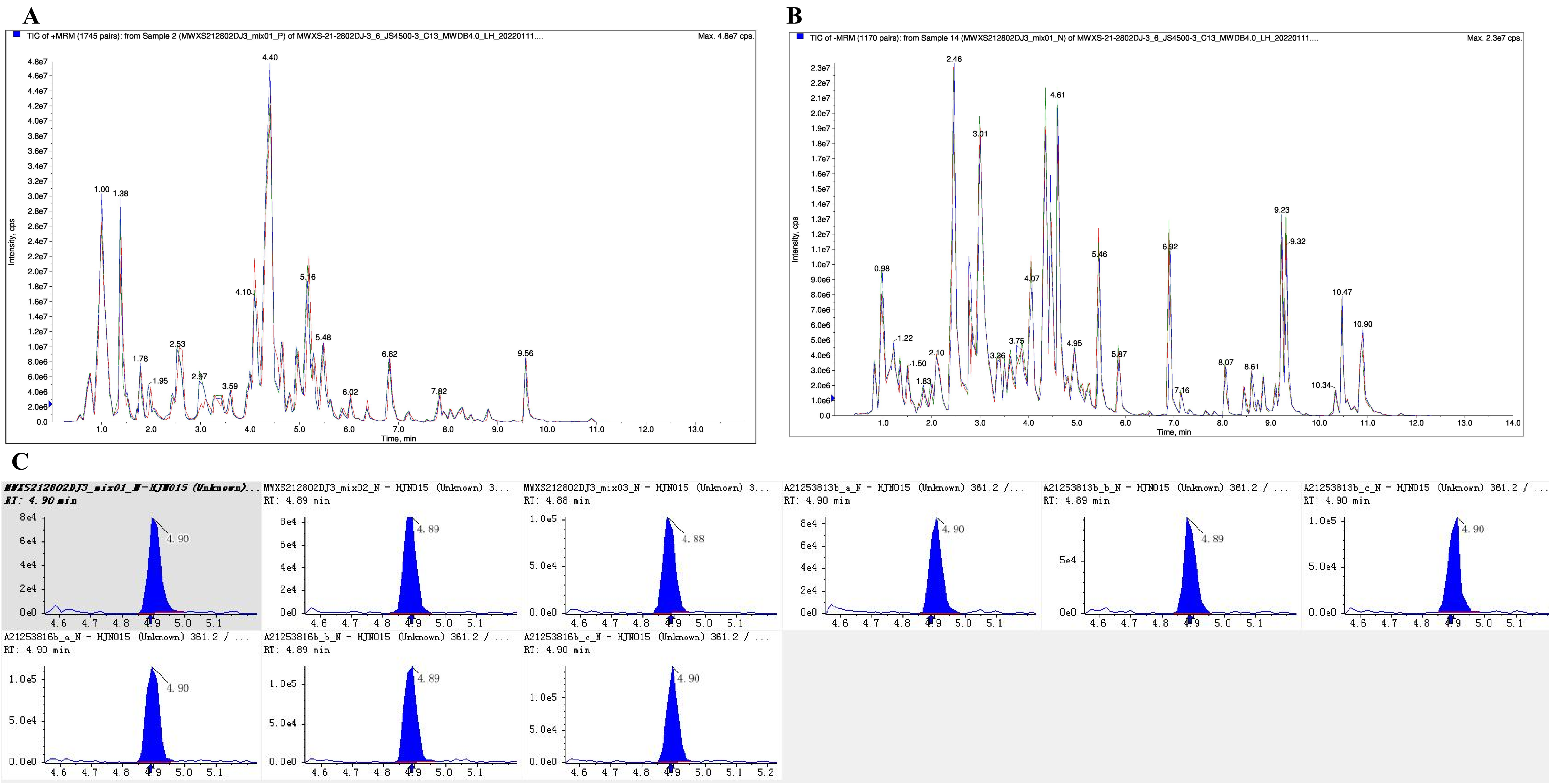

Figure S6. The TIC of LC-MS of mix sample with positive mode (A) and negative mode (B). The abscissa represents the retention time (min) of metabolite detection, and the ordinate represents the intensity of the ion current (cps: count per second). Integral correction diagram for quantitative analysis of some of the detected metabolites (C). The abscissa is the retention time (min) of the metabolite detection. The ordinate is the ion flow strength (CPS) detected by a metabolite ion. The peak area represents the relative content of the substance in the sample.

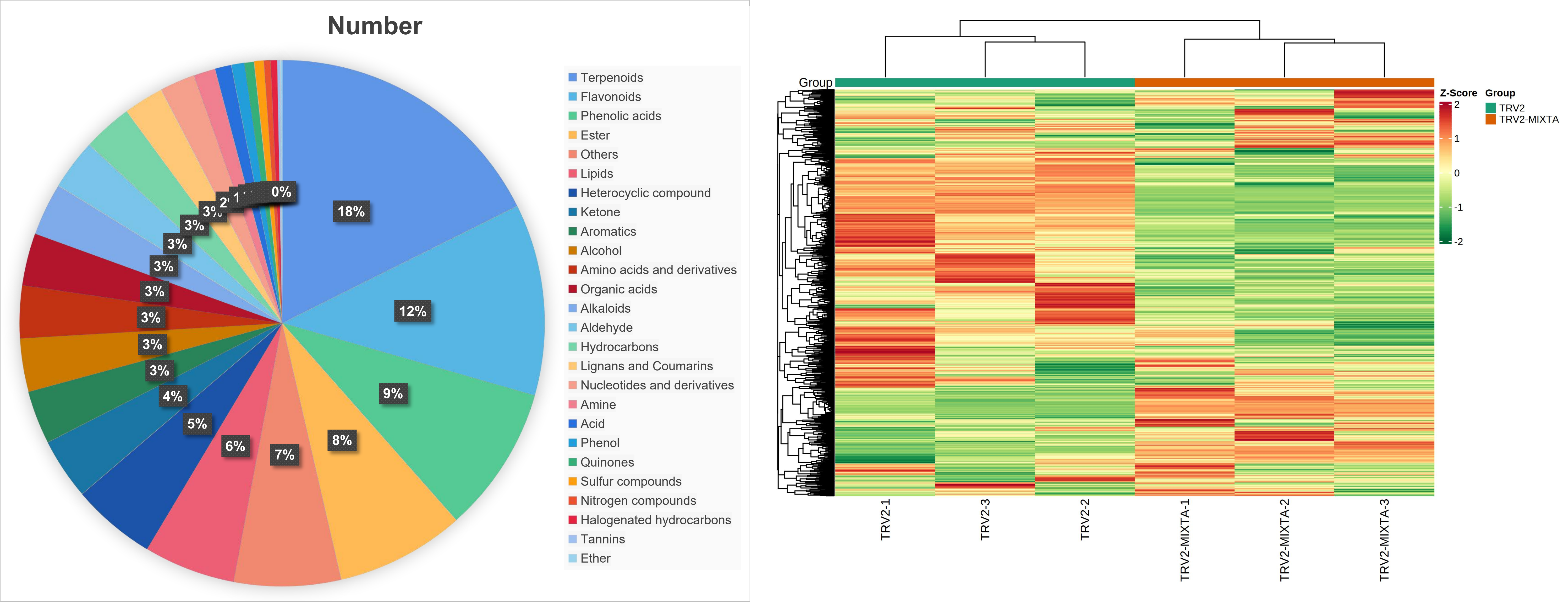

Figure S7. Classification of the 1705 identified metabolites (A) and hierarchical cluster analysis (HCA) results exposing metabolites variation between and within groups (B).

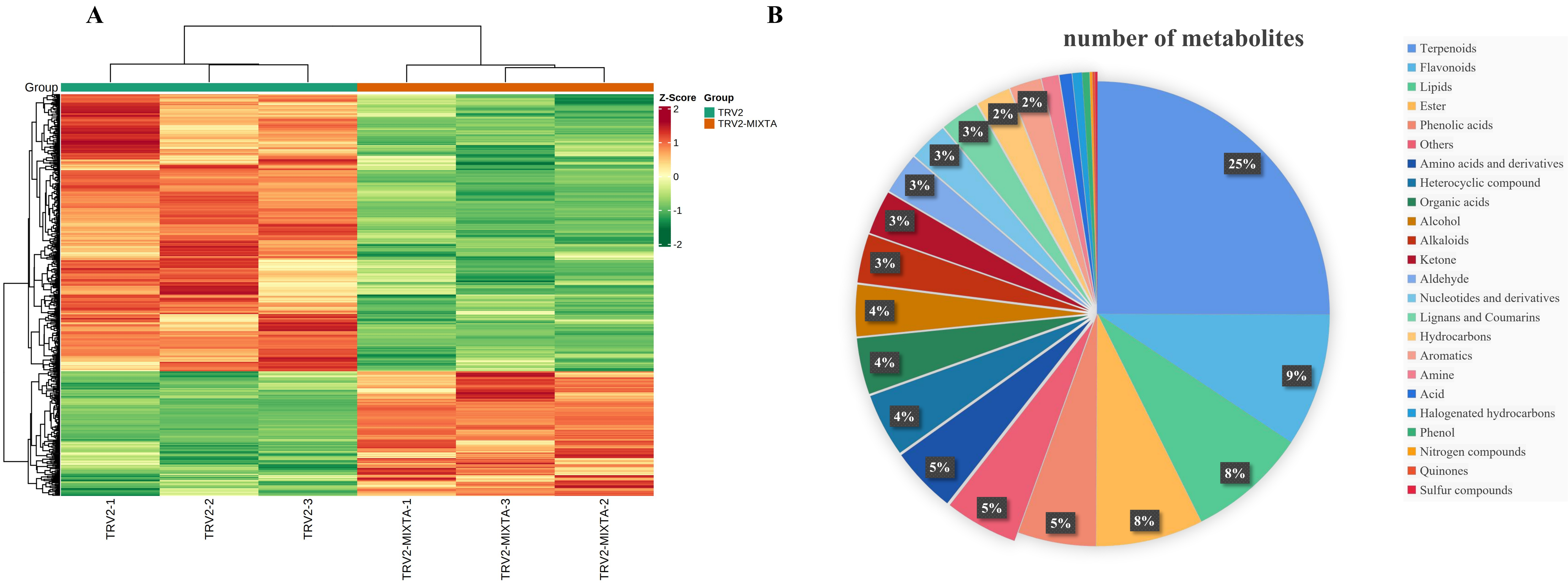

Figure S8. The heatmap of differential metabolites (A) and statistical analysis of differential metabolites (B) .

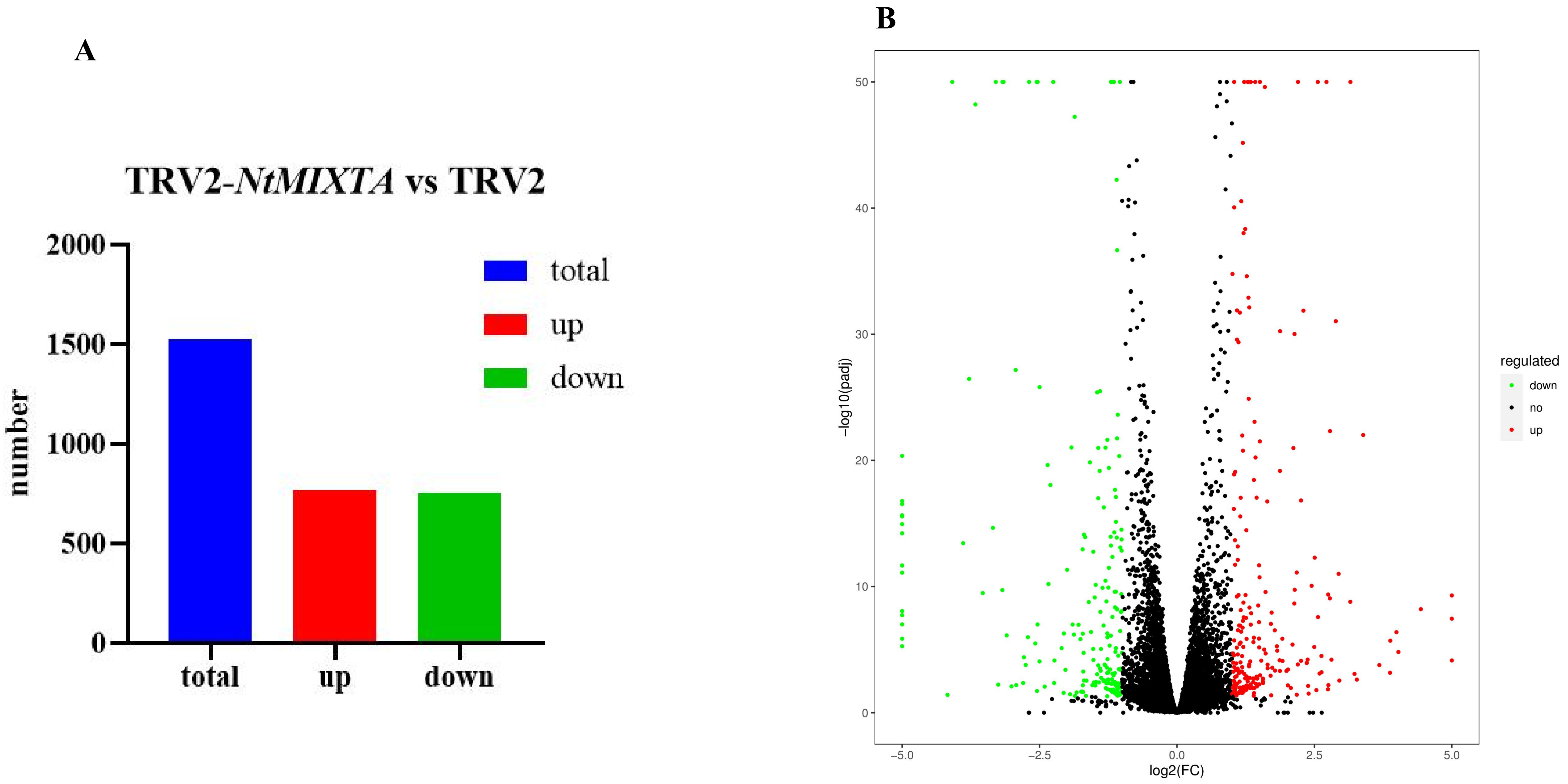

Figure S9. The differential expressed genes (DEGs) of TRV2-*NtMIXTA1* and TRV2 (A), and the volcano plot of DEGs (B).

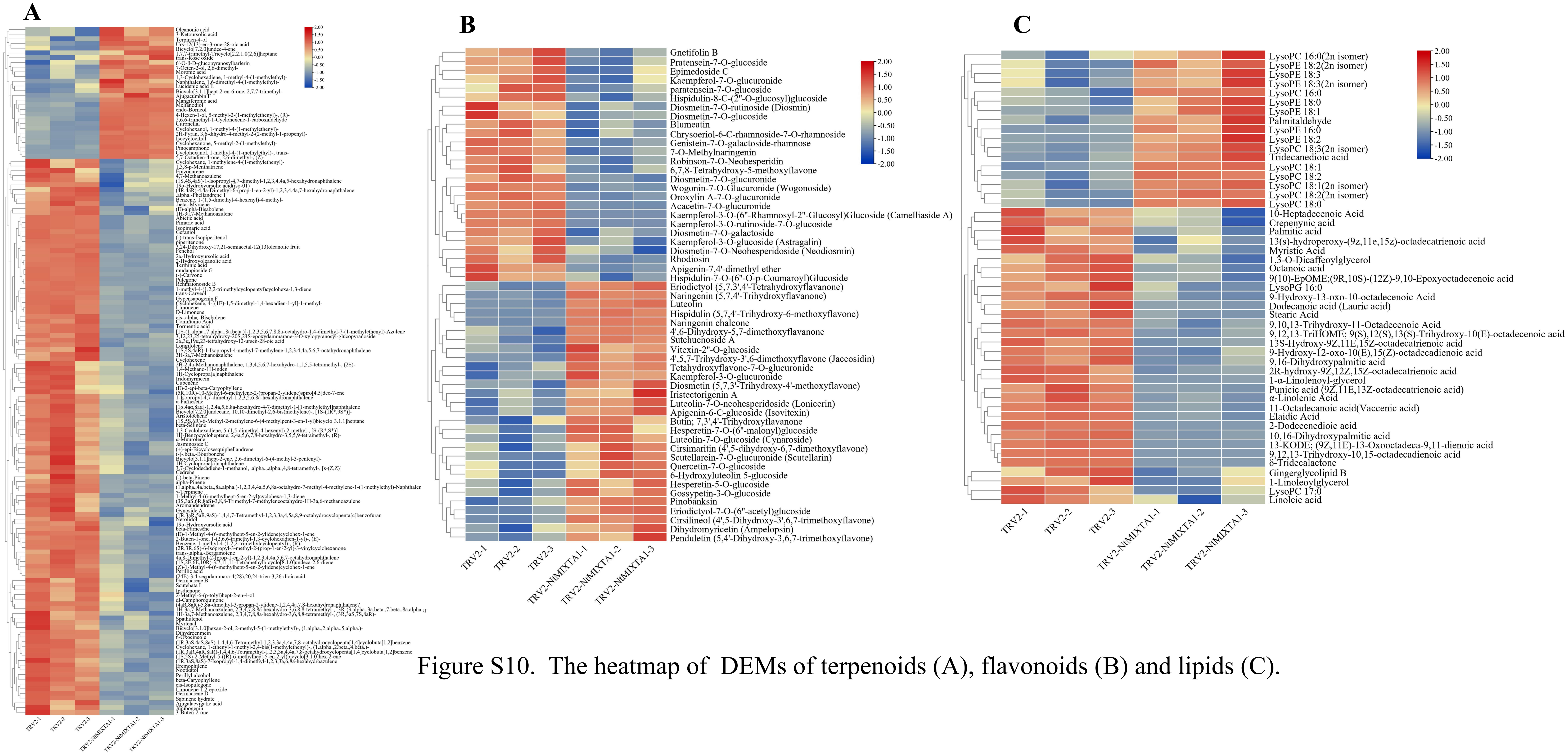

Figure S10. The heatmap of DEMs of terpenoids (A), flavonoids (B) and lipids (C).

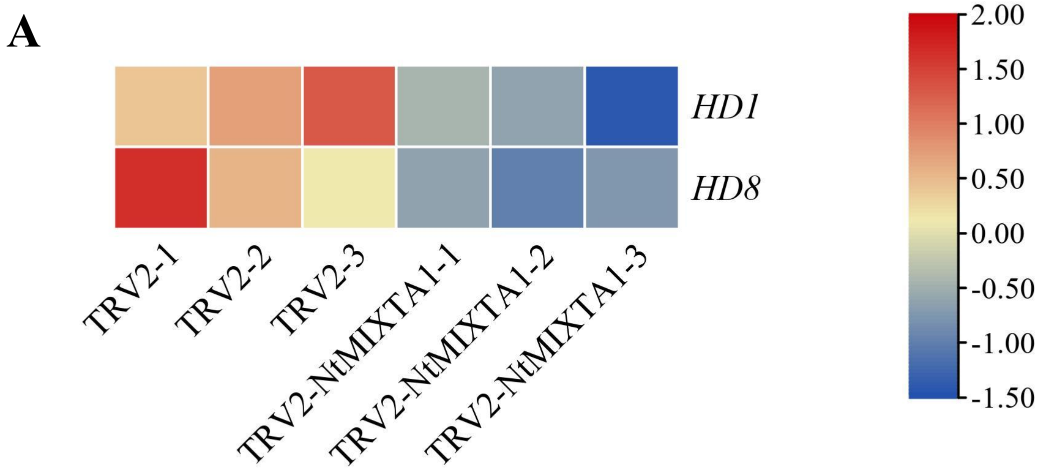

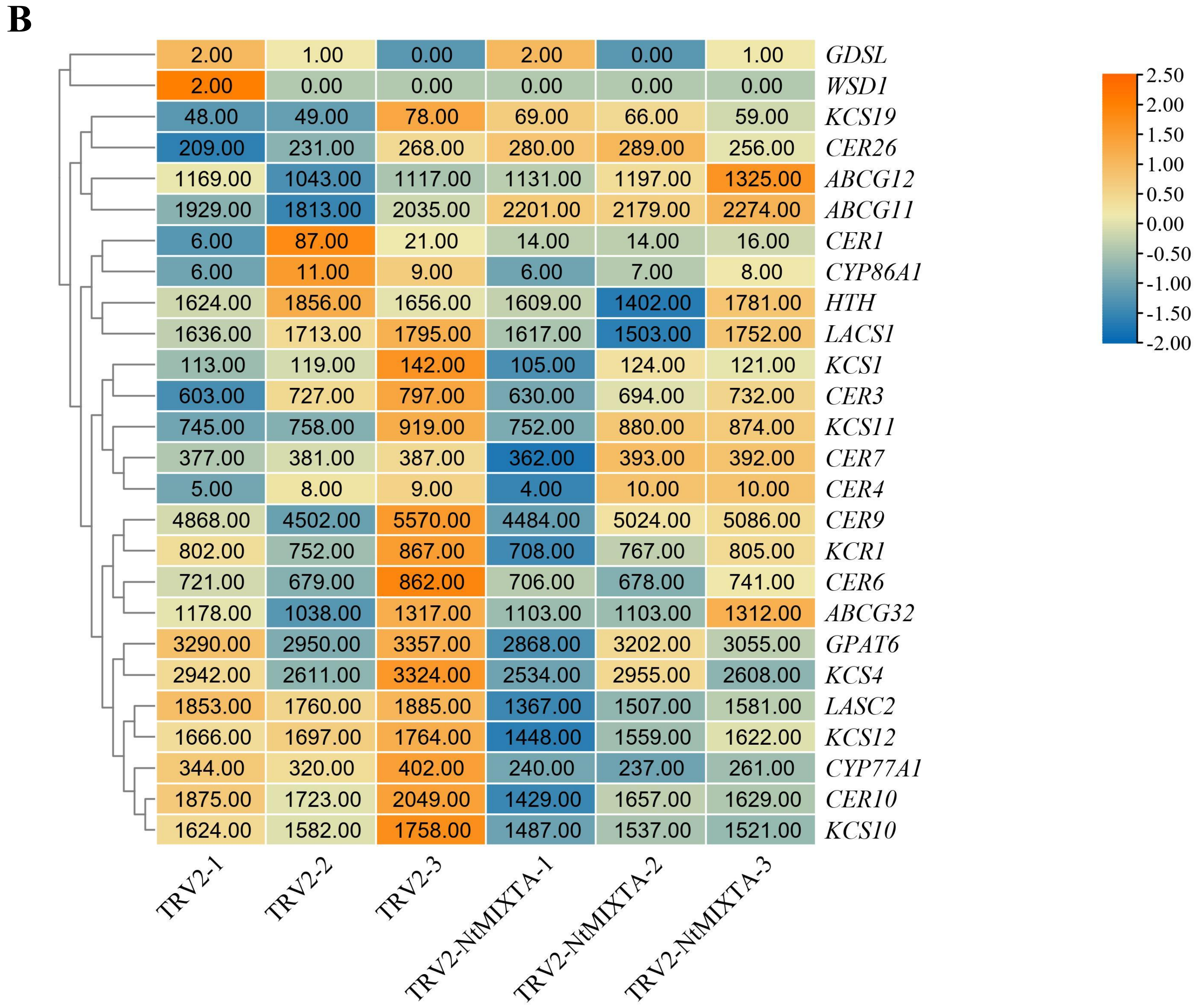

Figure S11. The expression of homologous gene of *AaHD1* and *AaHD8* (A). Hierarchical clustering analysis of genes involved in cuticle and wax biosynthesis (B).

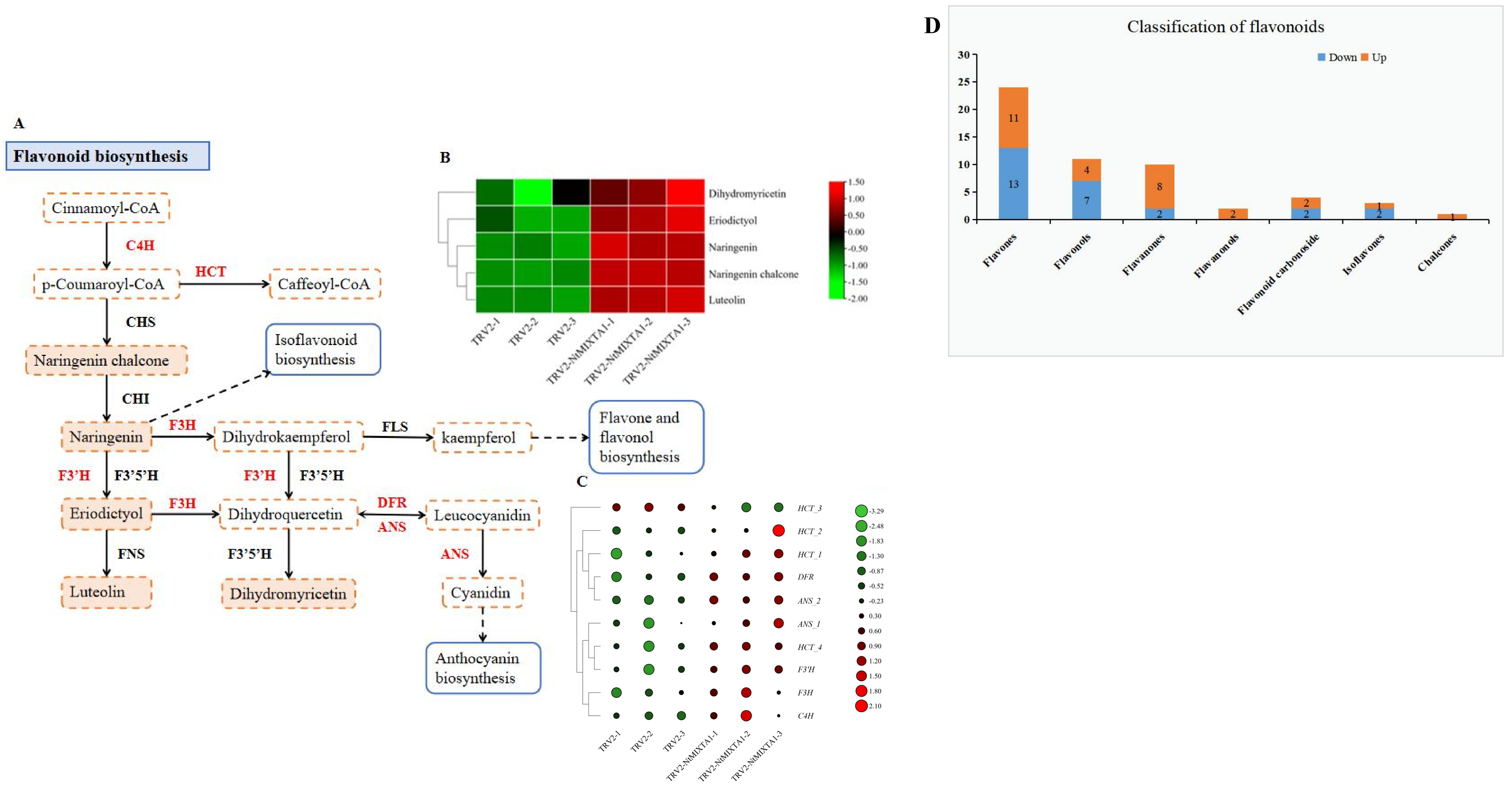

Figure S12. The biosynthesis of flavonoid biosynthesis (A), the heatmap of DEMs detected in this pathway (B) showing orange background color in pathway, and heatmap of DEGs this biosynthesis showing red color in pathway (C). Statistics on the structures of different flavonoids (D).
C4H, cinnamic acid 4-hydroxylase; CHS, chalcone synthase; HCT, shikimate O-hydroxycinnamoyltransferase; CHI, chalcone isomerase; F3H, flavonoid 3-hydroxylase; F3′H, flavonoid 3′-hydroxylase; F3′5′H, flavonoid 3′5′-hydroxylase; DFR, dihydroflavonol 4-reductase; ANS, anthocyanidin synthase; FLS, flavonol synthase.

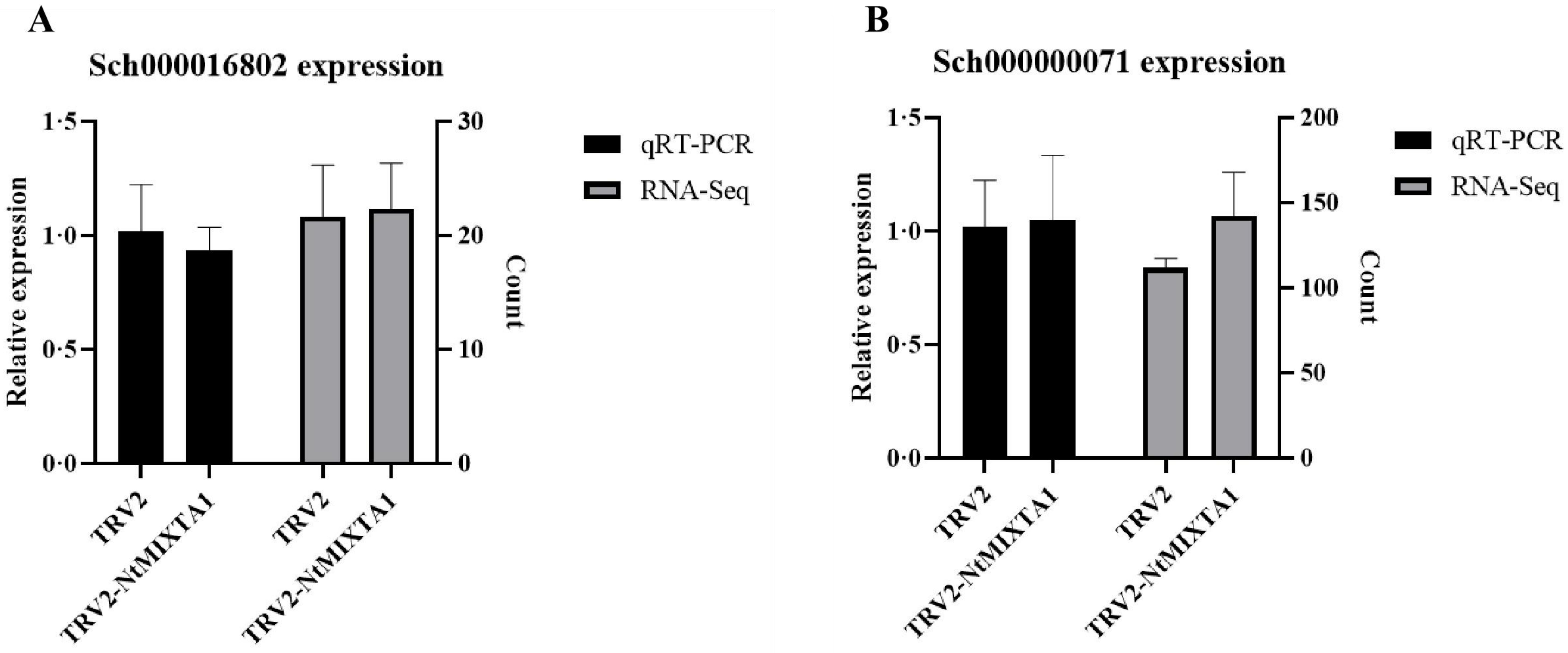

Figure S13. The gene expression of Sch000016802 (A) and Sch000000071 (B) of group TRV2 and TRV2-NtMIXTA in RNA-Seq and qRT-PCR.
