# Supplementary material for: Identification and characterization of a novel gene involved in glandular trichome development in Nepeta tenuifolia
Source: Front Plant Sci. 2022 Jul 29;13:936244. doi: 10.3389/fpls.2022.936244 (PMC9372485; doi:10.3389/fpls.2022.936244)
Supplement: Supplementary file 9 [file Data_Sheet_2.docx]

1. GC analysis of [essential](javascript:;) oil

The extracted volatile components were introduced into GC-FID instrument (7890A, Agilent Technologies) used a HP-5column (30 m x 320 μm x 0.25 μm; Agilent 19091J-413) with helium as the carrier gas. The initial oven temperature was initially 50℃ (held for 3 min), then raised to 100℃ at a rate of 3℃/min (held for 0 min), then raised to150°C at 5℃/min (held for 0 min), and finally raised to 220℃ at 10℃/min (held for 5 min) with post run for 5 min. Front inlet and detector temperatures were 250℃ with 8.8913 psi pressure, the inlet was operated in splitless mode, the injection volume was 1μL, and He carrier gasflow was set to 2.0 mL/min. The running time of whole method was 40.333 minutes. The hydrogen flow rate was 40 mL/min, the air flow rate was 400 mL/min, and the tail blowing flow rate was 28.3 mL/min.

1. LC-MS analysis

2.1 Sample preparation and extraction

  Biological samples are freeze-dried by vacuum freeze-dryer (Scientz-100F). The freeze-dried sample was crushed using a mixer mill (MM 400, Retsch) with a zirconia bead for 1.5 min at 30 Hz. Dissolve 100 mg of lyophilized powder with 1.2 mL 70% methanol solution, vortex 30 seconds every 30 minutes for 6 times in total, place the sample in a refrigerator at 4°C overnight. Following centrifugation at 12000 rpm for 10 min, the extracts were filtrated (SCAA-104, 0.22 μm pore size; ANPEL,Shanghai, China, <http://www.anpel.com.cn/>) before UPLC-MS/MS analysis.

2.2 UPLC Conditions

  The sample extracts were analyzed using an UPLC-ESI-MS/MS system (UPLC, SHIMADZU CBM30A, <https://www.shimadzu.com.cn/>; MS, Applied Biosystems 4500 Q TRAP, <https://www.thermofisher.cn/cn/zh/home/brands/applied-biosystems.html>). The analytical conditions were as follows, UPLC: column, Agilent SB-C18 (1.8 µm, 2.1 mm × 100 mm); The mobile phase was consisted of solvent A, pure water with 0.1% formic acid, and solvent B, acetonitrile with 0.1% formic acid. Sample measurements were performed with a gradient program that employed the starting conditions of 95% A, 5% B. Within 9 min, a linear gradient to 5% A, 95% B was programmed, and a composition of 5% A, 95% B was kept for 1 min. Subsequently, a composition of 95% A, 5.0% B was adjusted within 1.1 min and kept for 2.9 min. The flow velocity was set as 0.35 mL per minute; The column oven was set to 40°C; The injection volume was 4 μL. The effluent was alternatively connected to an ESI-triple quadrupole-linear ion trap (QTRAP)-MS.

2.3 ESI-Q TRAP-MS/MS

LIT and triple quadrupole (QQQ) scans were acquired on a triple quadrupole-linear ion trap mass spectrometer (Q TRAP), AB4500 Q TRAP UPLC/MS/MS System, equipped with an ESI Turbo Ion-Spray interface, operating in positive and negative ion mode and controlled by Analyst 1.6.3 software (AB Sciex). The ESI source operation parameters were as follows: ion source, turbo spray; source temperature 550°C; ion spray voltage (IS) 5500 V (positive ion mode)/-4500 V (negative ion mode); ion source gas I (GSI), gas II(GSII), curtain gas (CUR) were set at 50, 60, and 25.0 psi, respectively; the collision-activated dissociation(CAD) was high. Instrument tuning and mass calibration were performed with 10 and 100 μmol/L polypropylene glycol solutions in QQQ and LIT modes, respectively. QQQ scans were acquired as MRM experiments with collision gas (nitrogen) set to medium. DP and CE for individual MRM transitions was done with further DP and CE optimization. A specific set of MRM transitions were monitored for each period according to the metabolites eluted within this period.

2.4 Qualitative and quantitative analysis of metabolites on LC-MS platform

Independently establish database was generated based on multi-species, literature, partial criteria and retention index. The selected ion detection mode was carried out for accurate scanning, including certain RT and qualitative and quantitative ions. One quantitative ion and 2-3 qualitative ions were selected for each compound. All the ions to be detected in each group were detected at different times according to the peak order. If the retention time of the detected peaks was consistent with the standard reference and all the selected ions appeared in the sample quality spectrum after deducting the background. Quantitative ions were selected to integrate and correct chromatographic peaks to enhance the accuracy of quantification.

1. GC-MS analysis

3.1 Sample preparation and treatment

  Materials were harvested, weighted, immediately frozen in liquid nitrogen, and stored at -80°C until needed. Samples were ground to a powder in liquid nitrogen.

  1 g (1 mL) of the powder was transferred immediately to a 20 mL head-space vial (Agilent, Palo Alto, CA, USA), containing NaCl saturated solution, to inhibit any enzyme reaction. The vials were sealed using crimp-top caps with TFE-silicone headspace septa (Agilent). At the time of SPME analysis, each vial was placed in 60°C for 5 min, then a 120 µm DVB/CWR/PDMS fibre (Agilent) was exposed to the headspace of the sample for 15 min at 100°C.

3.2 GC-MS conditions

After sampling, desorption of the VOCs from the fibre coating was carried out in the injection port of the GC apparatus (Model 8890; Agilent) at 250°C for 5 min in the splitless mode. The identification and quantification of VOCs was carried out using an Agilent Model 8890 GC and a 7000D mass spectrometer (Agilent), equipped with a 30 m × 0.25 mm × 0.25 μm DB-5MS (5% phenyl-polymethylsiloxane) capillary column. Helium was used as the carrier gas at a linear velocity of 1.2 mL/min. The injector temperature was kept at 250°C and the detector at 280°C. The oven temperature was programmed from 40°C (3.5 min), increasing at 10°C/min to 100°C, at 7°C/min to 180°C, at 25°C/min to 280°C, hold for 5 min. Mass spectra was recorded in electron impact (EI) ionisation mode at 70 eV. The quadrupole mass detector, ion source and transfer line temperatures were set, respectively, at 150, 230 and 280°C. The MS was selected ion monitoring (SIM) mode was used for the identification and quantification of analytes.

Metabolite Identification and Quantification

3.3 Qualitative and quantitative analysis of metabolites on LC-MS platform

Based on MWDB (Metware Database), material was qualitative according to secondary spectrum information. Isotope signals, repeated signals containing K+, Na+ and NH4+, as well as repeated signals of fragments of other substances with larger molecular weight were removed during analysis.

The metabolites were quantified by multiple reaction monitoring (MRM) using triple quadrupole mass spectrometry. In MRM mode, the quadrupole firstly screened the precursor ions (parent ions) of the target substance, and excluded the corresponding ions of other molecular weight substances to eliminate the interference initially. After ionization induced by the impact chamber, the precursor ions break and form many fragments, and then a characteristic fragment ion is selected through the triple quadrupole filtration to exclude the interference of non-target ions, so that the quantification is more accurate and repeatability is better. After obtaining the metabolite spectrum analysis data of different samples, the mass spectrum peaks of all substances were integrated with the peak area, and the mass spectrum peaks of the same metabolite in different samples were integrated with the correction.

1. Metabolites Data anaysis

4.1 PCA

  Unsupervised PCA (principal component analysis) was performed by statistics function prcomp within R (www.r-project.org). The data was unit variance scaled before unsupervised PCA.

4.2 Hierarchical Cluster Analysis and Pearson Correlation Coefficients

  The HCA (hierarchical cluster analysis) results of samples and metabolites were presented as heatmaps with dendrograms, while pearson correlation coefficients (PCC) between samples were caculated by the cor function in R and presented as only heatmaps. Both HCA and PCC were carried out by R package ComplexHeatmap. For HCA, normalized signal intensities of metabolites (unit variance scaling) are visualized as a color spectrum.

4.3 Differential metabolites selected

  Significantly regulated metabolites between groups were determined by VIP ≥ 1 and p value < 0.05. VIP values were extracted from OPLS-DA result, which also contain score plots and permutation plots, was generated using R package MetaboAnalystR. The data was log transform (log2) and mean centering before OPLS-DA. In order to avoid overfitting, a permutation test (200 permutations) was performed.

4.4 KEGG annotation and enrichment analysis

  Identified metabolites were annotated using KEGG Compound database (<http://www.kegg.jp/kegg/compound/>), annotated metabolites were then mapped to KEGG Pathway database (<http://www.kegg.jp/kegg/pathway.html>). Pathways with significantly regulated metabolites mapped to were then fed into MSEA (metabolite sets enrichment analysis), their significance was determined by hypergeometric test’s p-values.

1. RNA-seq

5.1 RNA quantification and qualification

RNA degradation and contamination was monitored on 1% agarose gels. RNA purity was checked using the NanoPhotometer® spectrophotometer (IMPLEN, CA, USA). RNA concentration was measured using Qubit® RNA Assay Kit in Qubit®2.0 Flurometer (Life Technologies, CA, USA). RNA integrity was assessed using the RNA Nano 6000 Assay Kit of the Bioanalyzer 2100 system (Agilent Technologies, CA, USA).

5.2 Library preparation for Transcriptome sequencing

A total amount of 1 µg RNA per sample was used as input material for the RNA sample preparations. Sequencing libraries were generated using NEBNext® UltraTM RNA Library Prep Kit for Illumina® (NEB, USA) following manufacturer’s recommendations and index codes were added to attribute sequences to each sample. Briefly, mRNA was purified from total RNA using poly-T oligo attached magnetic beads. Fragmentation was carried out using divalent cations under elevated temperature in NEBNext First Strand Synthesis Reaction Buffer(5X). First strand cDNA was synthesized using random hexamer primer and M-MuLV Reverse Transcriptase(RNase H-). Second strand cDNA synthesis was subsequently performed using DNA Polymerase I and RNase H. Remaining overhangs were converted into blunt ends via exonuclease/polymerase activities. After adenylation of 3’ ends ofDNA fragments, NEBNext Adaptor with hairpin loop structure were ligated to prepare for

hybridization. In order to select cDNA fragments of preferentially 250~300 bp in length, the library fragments were purified with AMPure XP system (Beckman Coulter, Beverly, USA). Then 3 µl USER Enzyme (NEB, USA) was used with size-selected, adaptor-ligated cDNA at 37°C for 15 min followed by 5 min at 95 °C before PCR. Then PCR was performed with Phusion High-Fidelity DNA polymerase,

Universal PCR primers and Index (X) Primer. Atlast, PCR products were purified (AMPure XP system) and library quality was assessed on the Agilent Bioanalyzer 2100 system.

5.3 Clustering and sequencing

The clustering of the index-coded samples was performed on a cBot Cluster Generation System using TruSeq PE Cluster Kit v3-cBot-HS (Illumia) according to the manufacturer’s instructions. After clustervgeneration, the library preparations were sequenced on an Illumina Hiseq platform and 125 bp/150 bp paired-end reads were generated.

5.4 Data Analysis

5.4.1 Data quality control

Use fastp v 0.19.3 to filter the original data, mainly to remove reads with adapters; when the N content in any sequencing reads exceeds 10% of the base number of the reads, remove the paired reads; when any sequencing reads When the number of low-quality (Q<=20) bases contained in reads exceeds 50% of the bases of the reads, this paired reads will be removed. All subsequent analyses are based on clean reads.

5.4.2 Reads mapping to the reference genome

Download the reference genome and its annotation files from the designated website, use HISAT v2.1.0 to construct the index, and compare clean reads to the reference genome.

5.4.3New transcript prediction

Use StringTie v1.3.4d for new gene prediction. StringTie applies network streaming algorithms and optional de novo to splice transcripts. Compared with Cufflinks and other software, StringTie can splice a more complete and accurate transcript, and the splicing speed is faster.

5.4.4 Quantification of gene expression levels

Use featureCounts v1.6.2 to calculate the gene alignment, and then calculate the FPKM of each gene based on the gene length. FPKM is currently the most commonly used method to estimate gene expression levels.

5.4.5 Difference analysis

DESeq2 v1.22.1 was used to analyze the differential expression between the two groups, and the Pvalue was corrected using the Benjamini & Hochberg method. The corrected P value < 0.5 and |log2foldchange| ≥ 1are used as the threshold for significant difference expression.

5.4.6 Differential gene enrichment analysis

The enrichment analysis is performed based on the hypergeometric test. For KEGG, the hypergeometric distribution test is performed with the unit of pathway; for GO, it is performed based on the GO term.

5.4.7 Differential gene protein interaction analysis

The protein interaction analysis of differentially expressed genes is based on the STRING database of known and predicted protein-protein interactions. For the species existing in the database, we construct the network by extracting the target gene list from the database; otherwise, use blast v2.7.1+ to compare the target gene sequence with the selected reference protein sequence, and then according to the selected reference species Know the interaction to build a network.
