# Supplementary material for: Identification and characterization of a novel gene involved in glandular trichome development in Nepeta tenuifolia
Source: Front Plant Sci. 2022 Jul 29;13:936244. doi: 10.3389/fpls.2022.936244 (PMC9372485; doi:10.3389/fpls.2022.936244)

Biosynthesis related to terpenoids：

ko00900: Terpenoid backbone biosynthesis；

ko00902: Monoterpenoid biosynthesis;

ko00904: Diterpenoid biosynthesis;

ko00909: Sesquiterpenoid and triterpenoid biosynthesis;


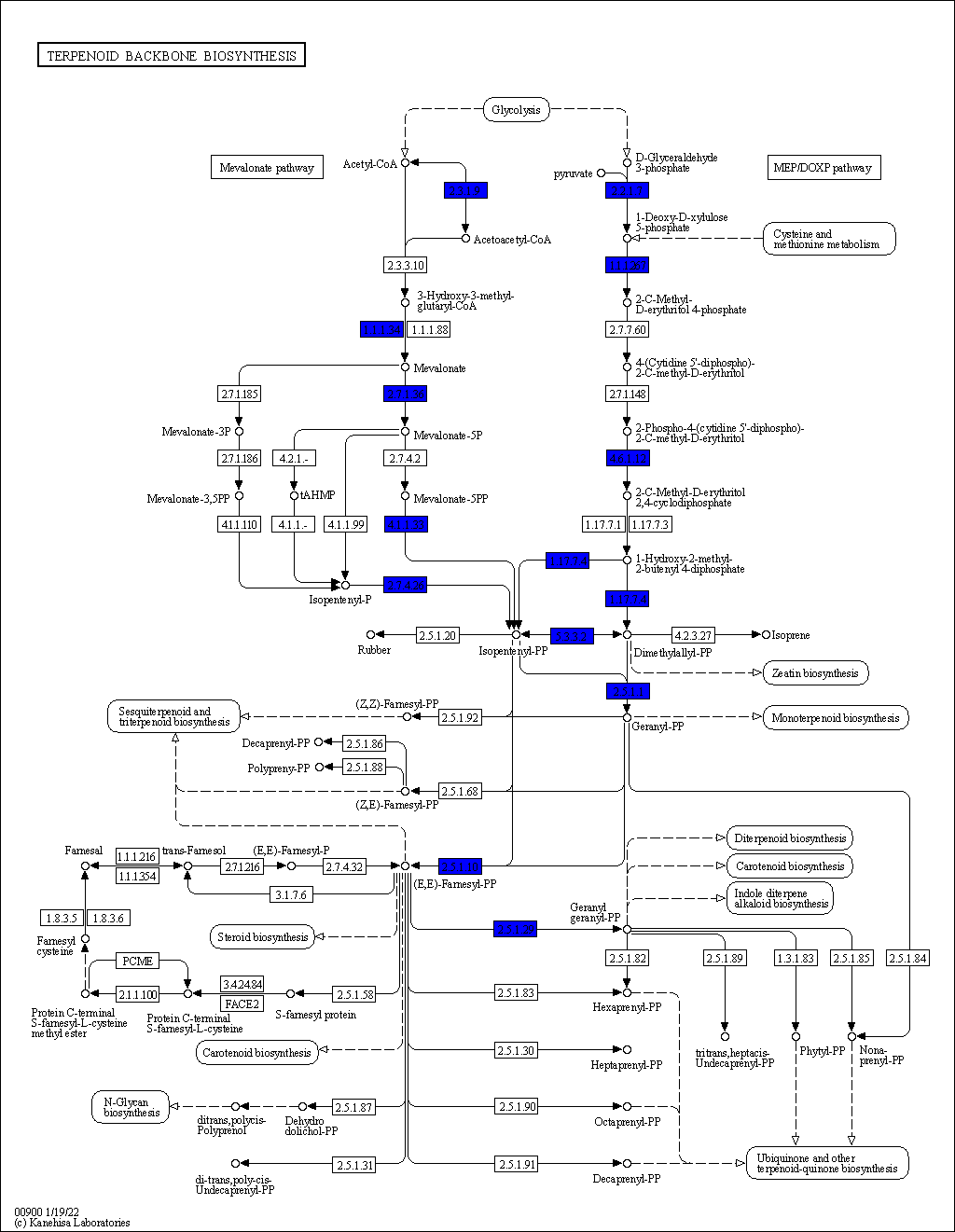


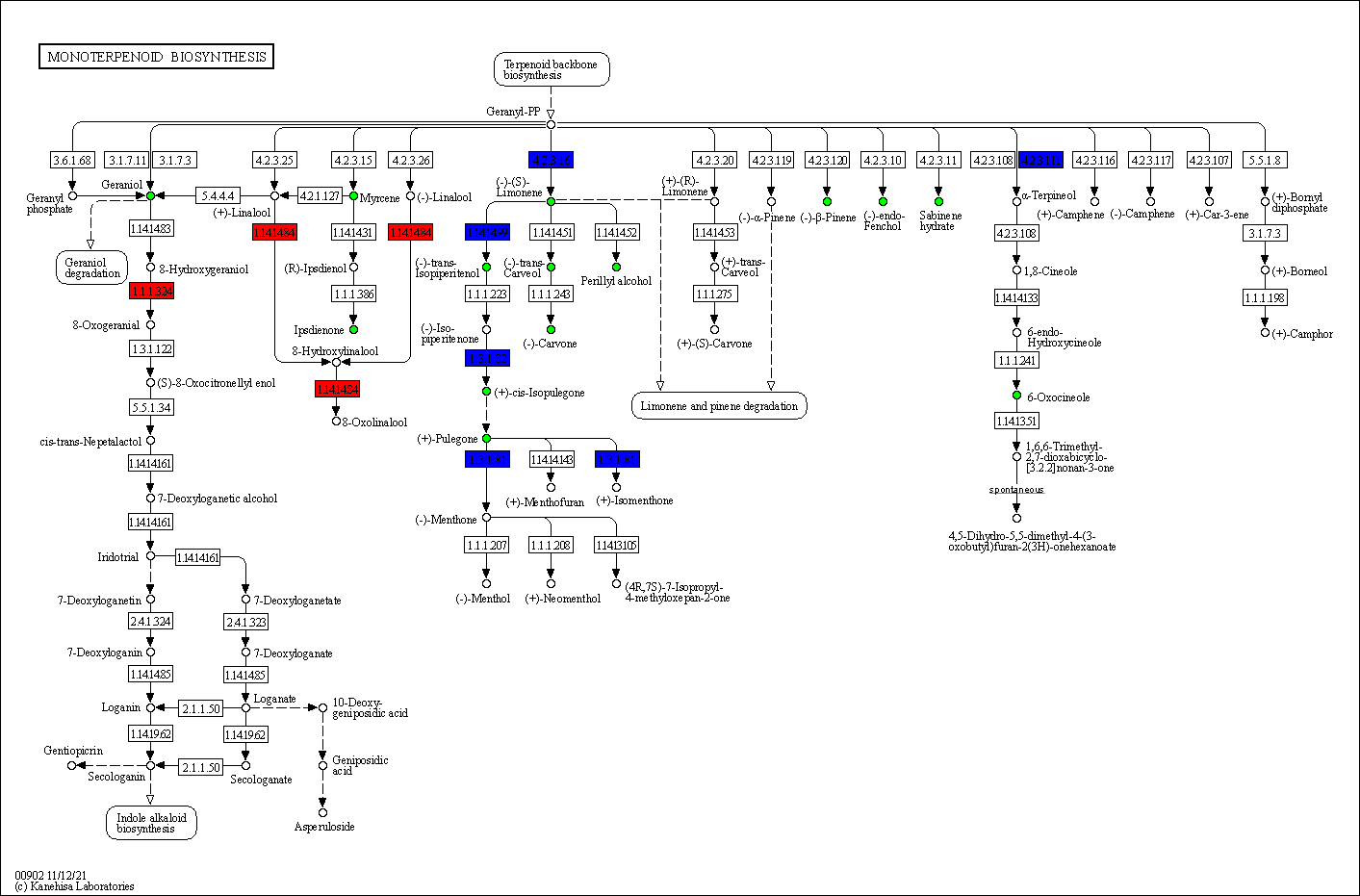

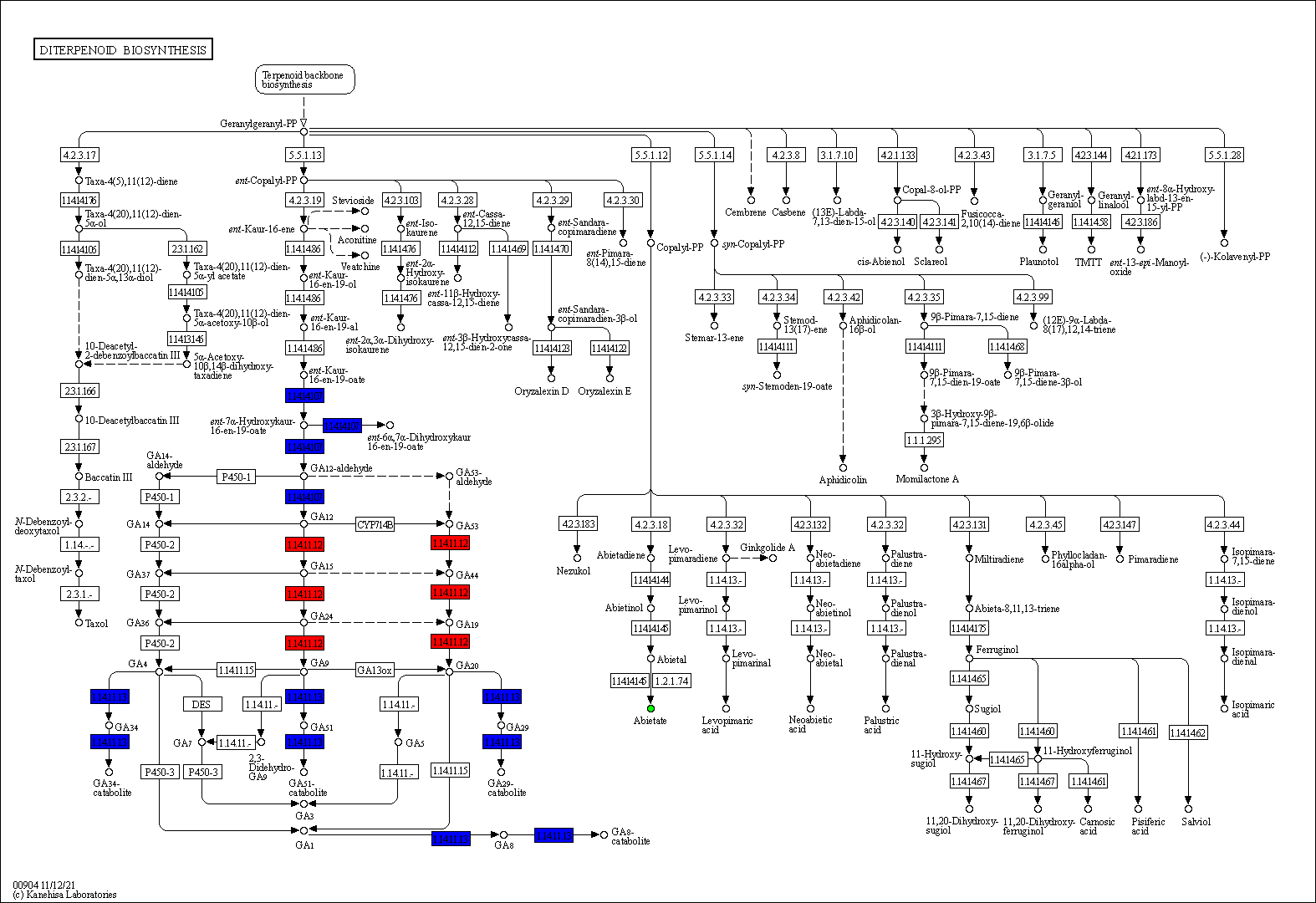


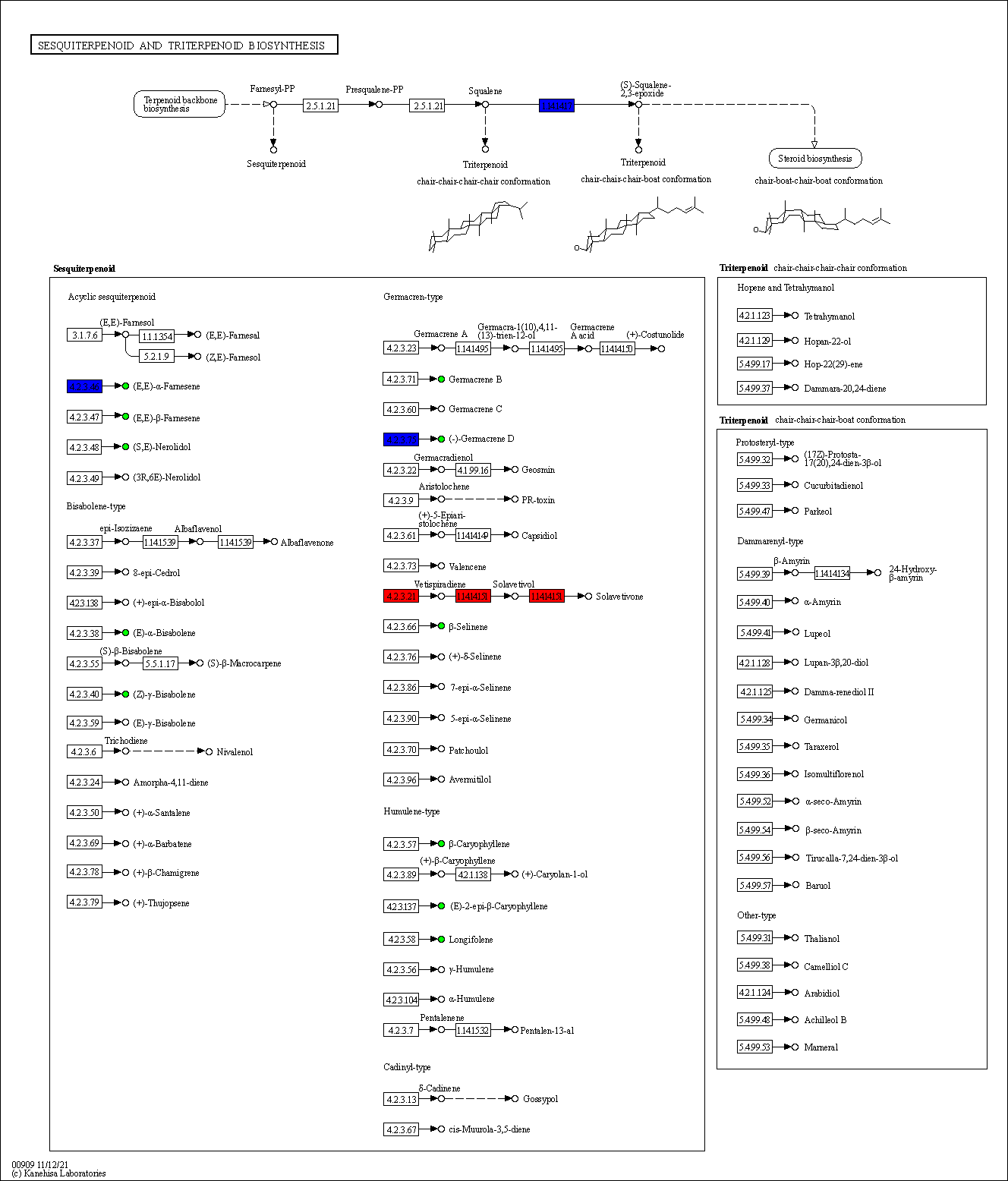


Biosynthesis related to flavonoids

ko00941: Flavonoid biosynthesis;

ko00944: Flavone and flavonol biosynthesis；

ko00942: Anthocyanin biosynthesis;

ko00943: Isoflavonoid biosynthesis;


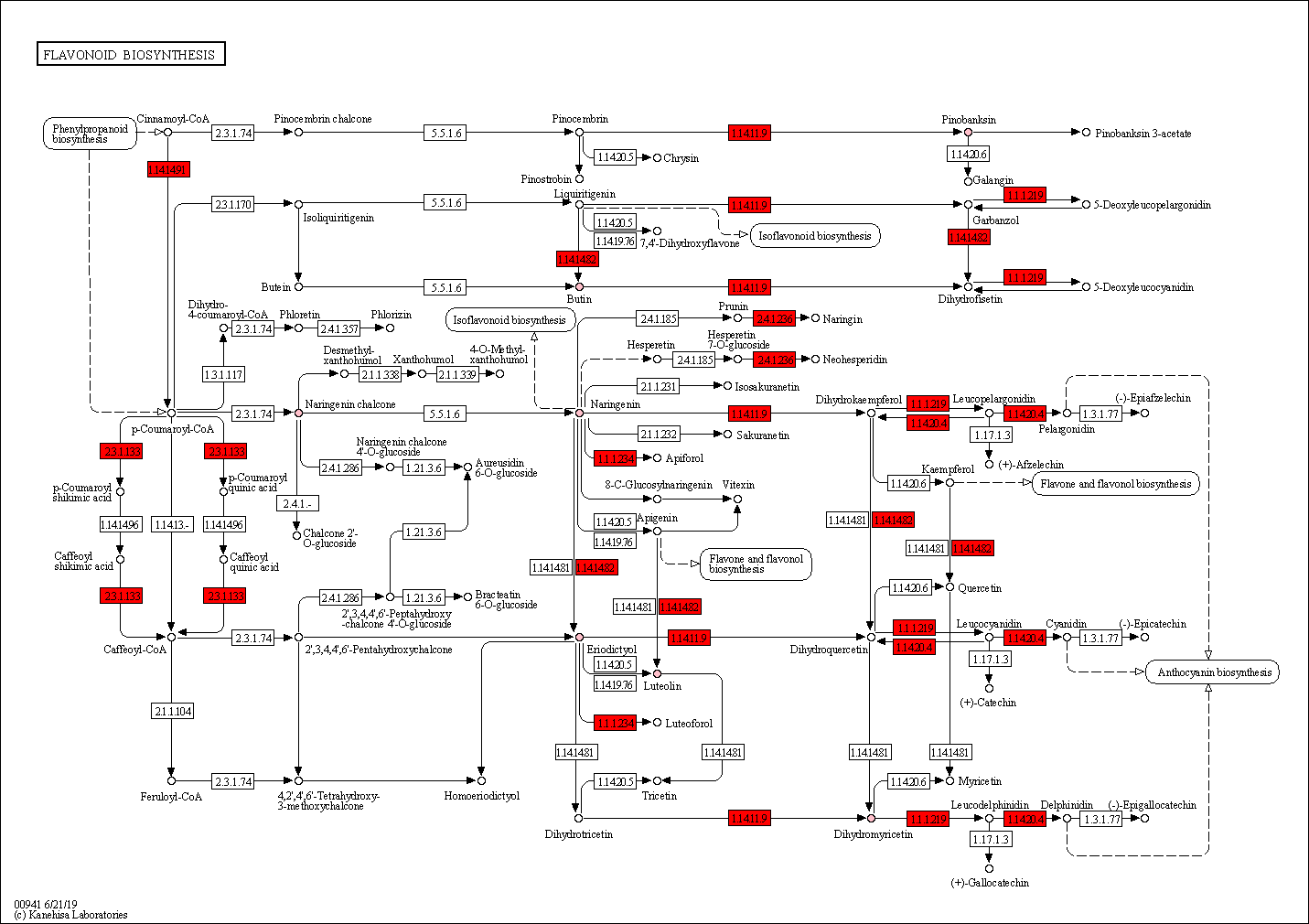


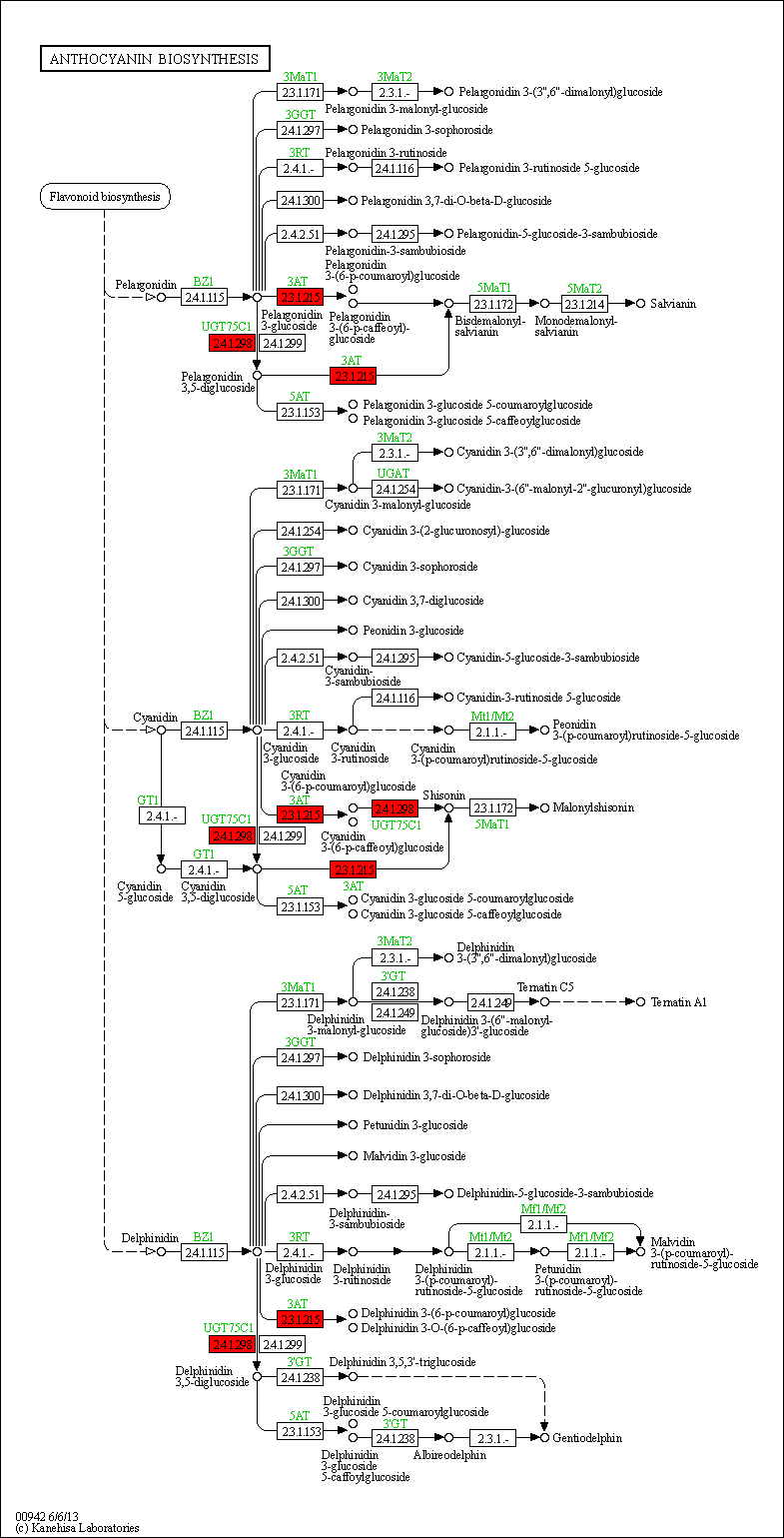


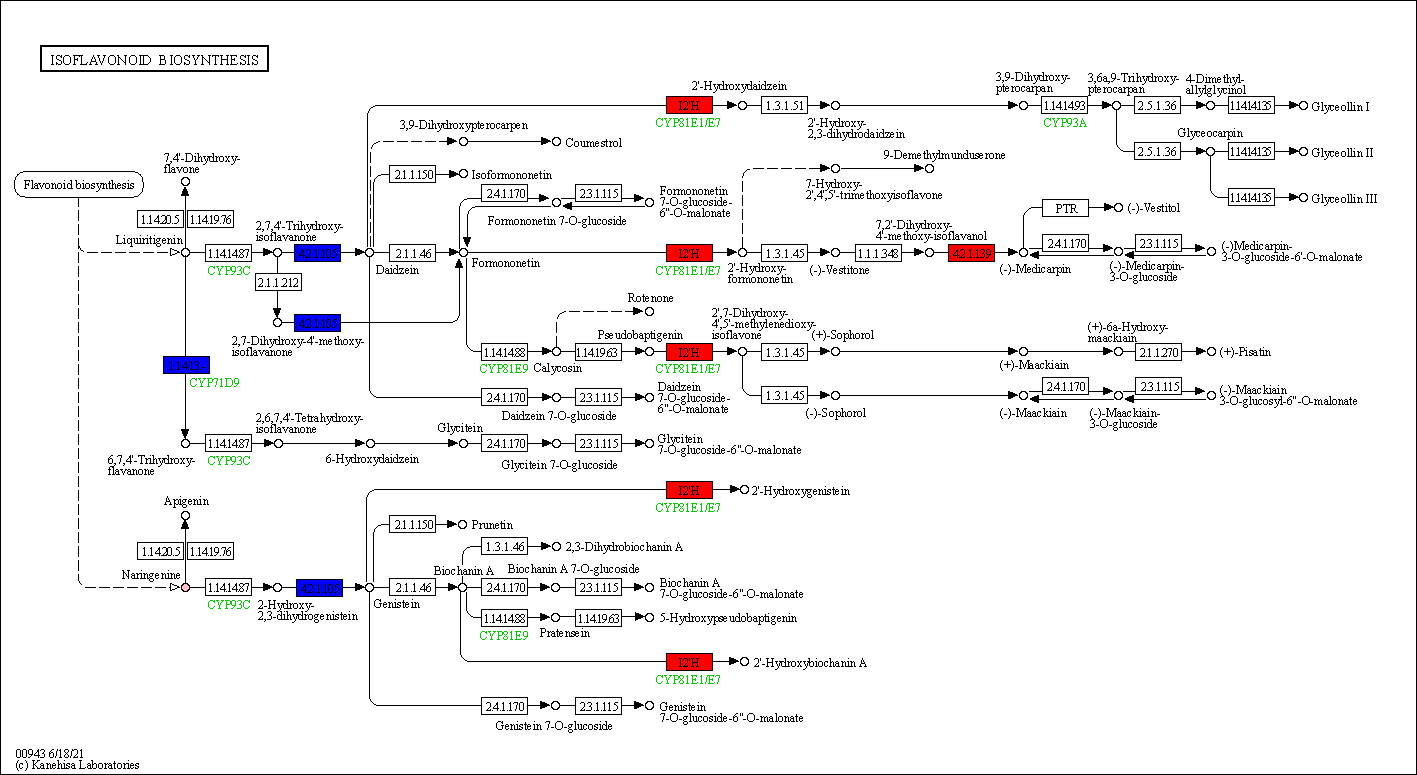


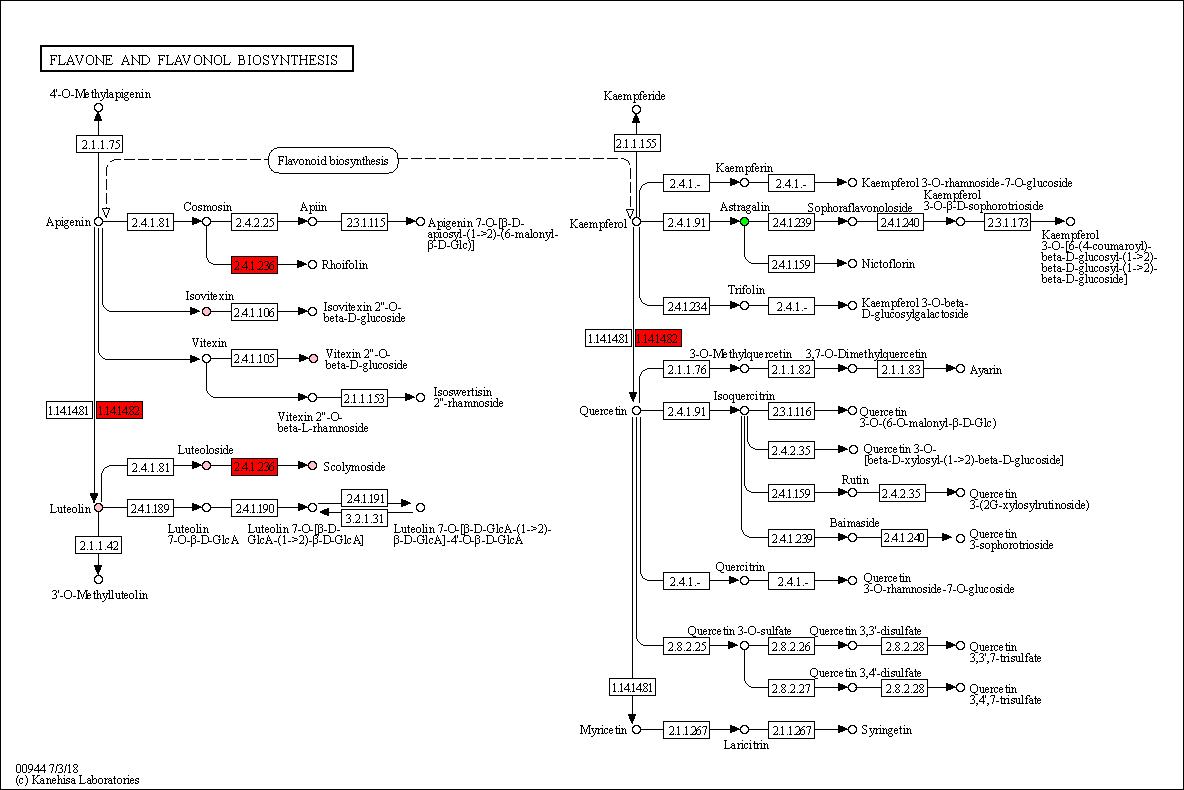


Biosynthesis of lipids

ko00061: Fatty acid biosynthesis;

ko00062: Fatty acid elongation;

ko00071: Fatty acid degradation；

ko00073: Cutin, suberine and wax biosynthesis

ko01040: Biosynthesis of unsaturated fatty acids;

ko01212: Fatty acid metabolism；

ko00592: alpha-Linolenic acid metabolism

ko00591: Linoleic acid metabolism


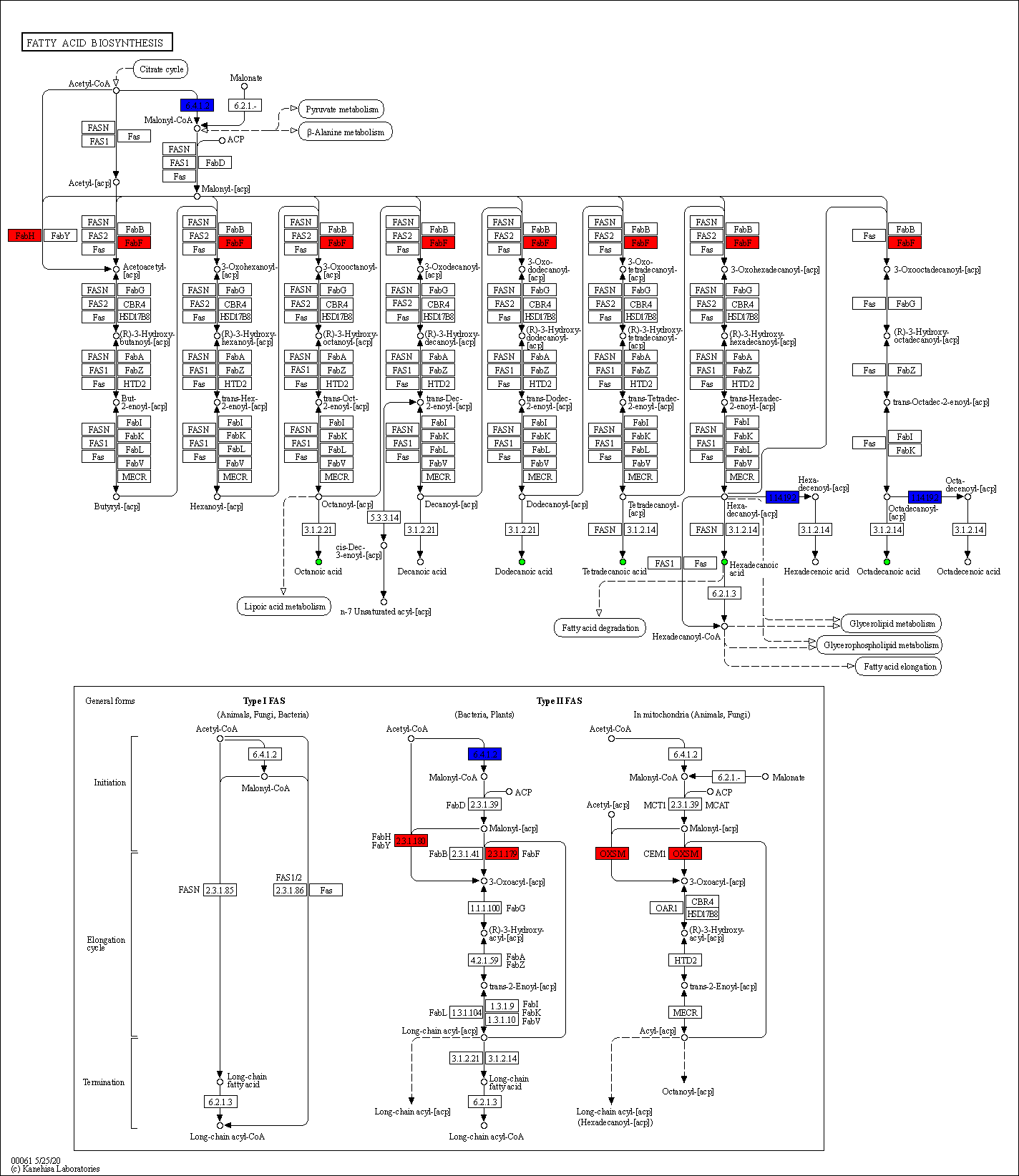


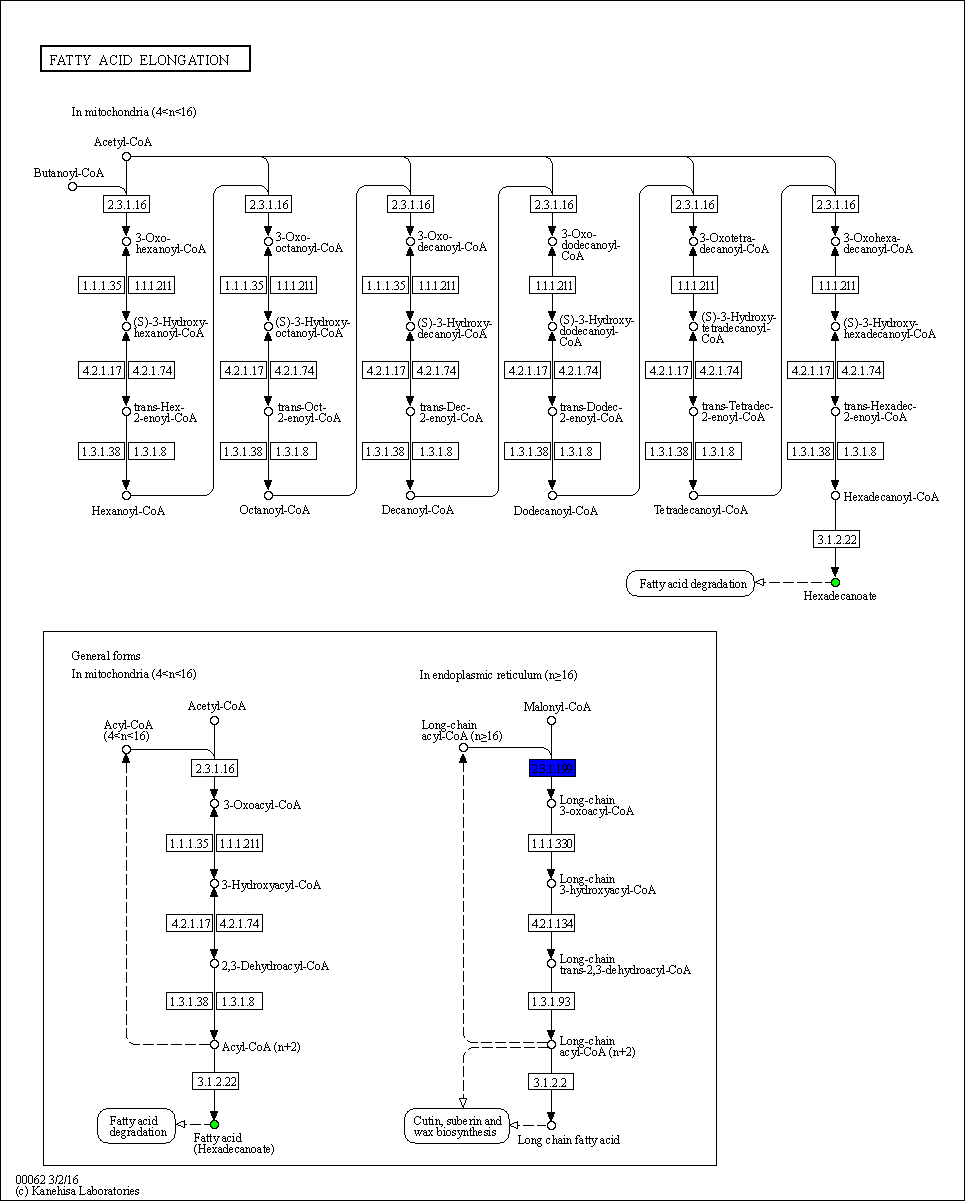


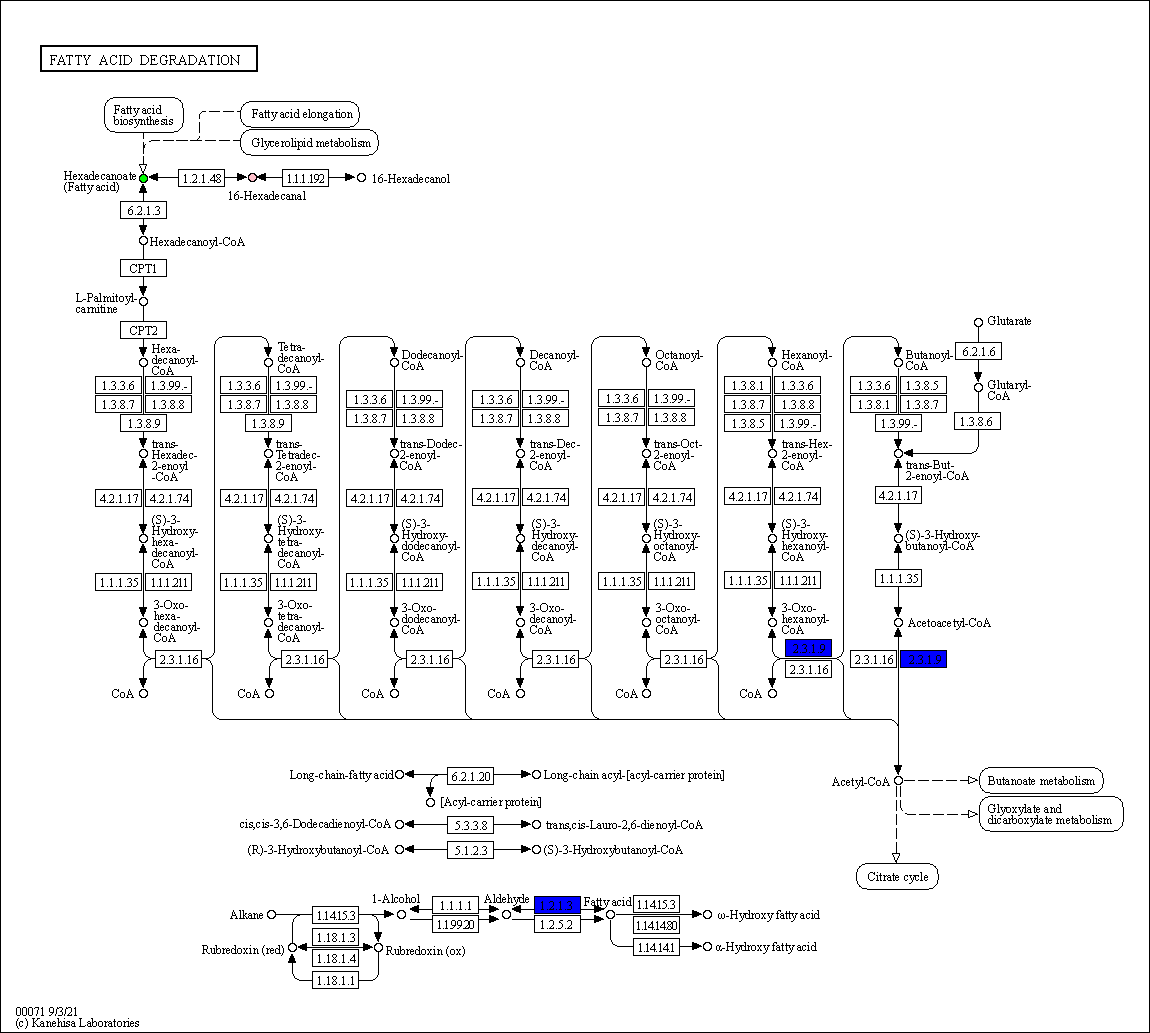

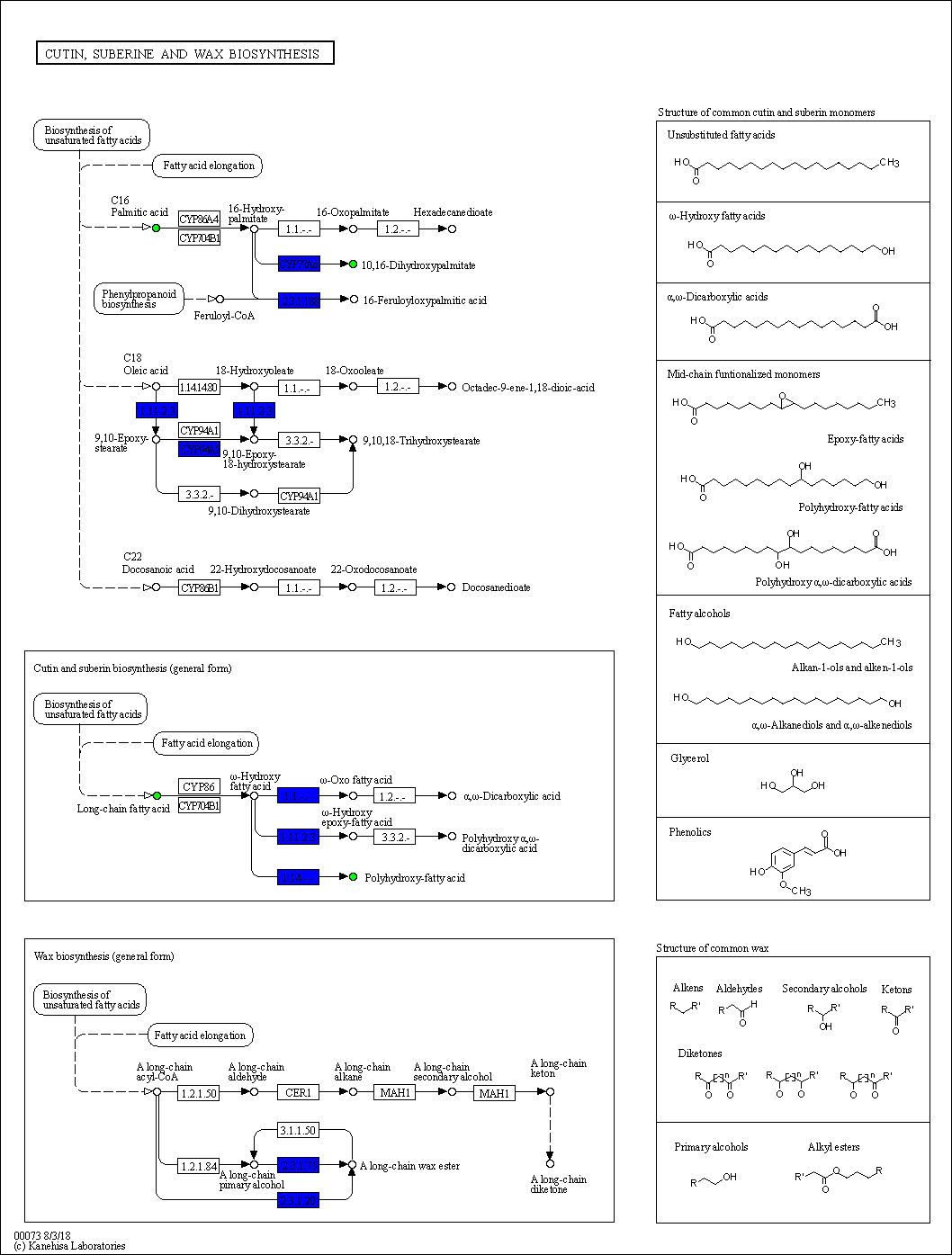

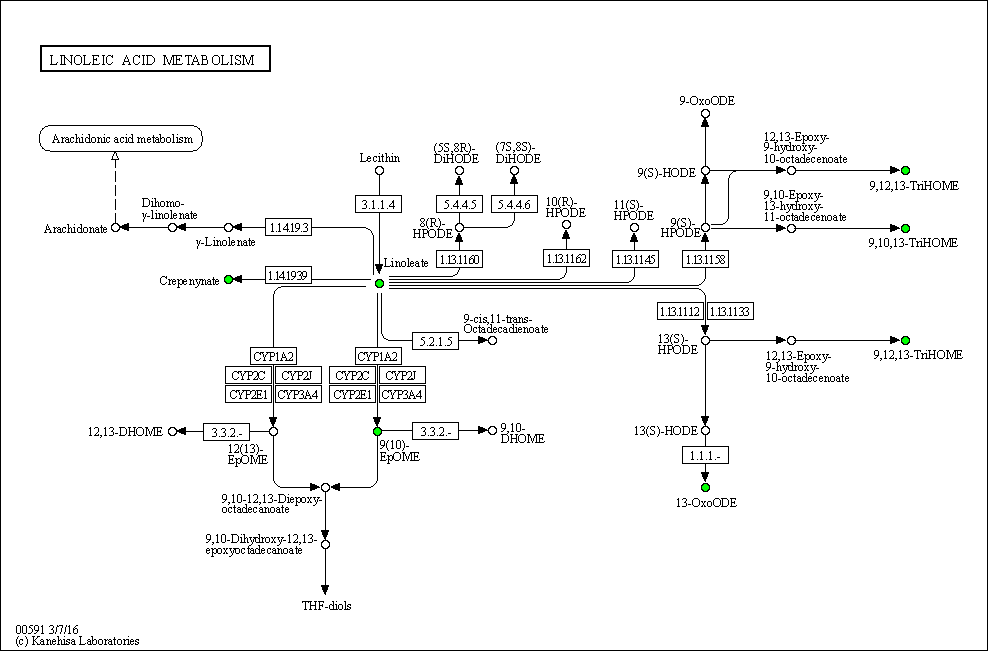


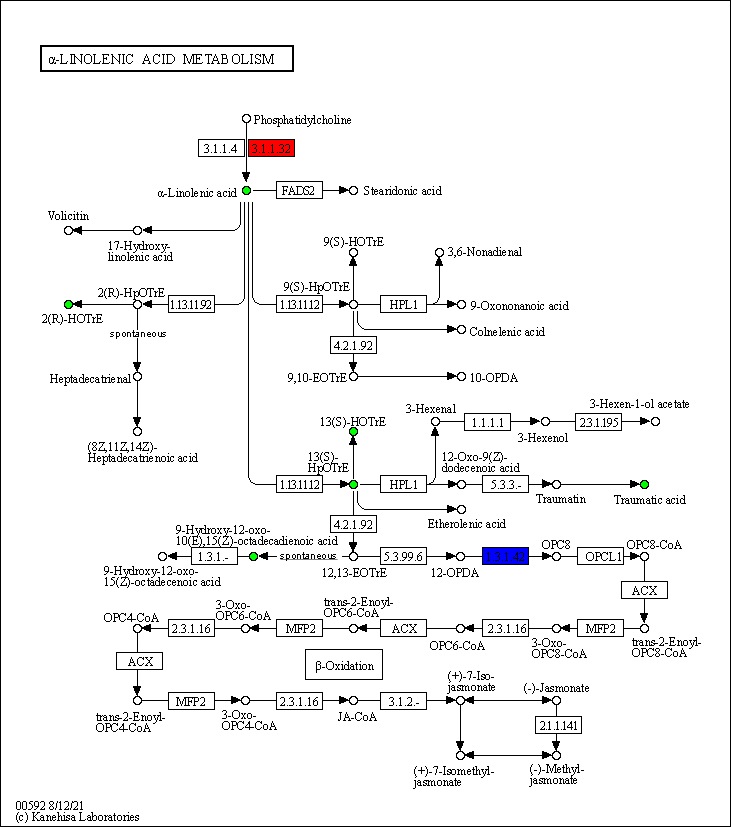


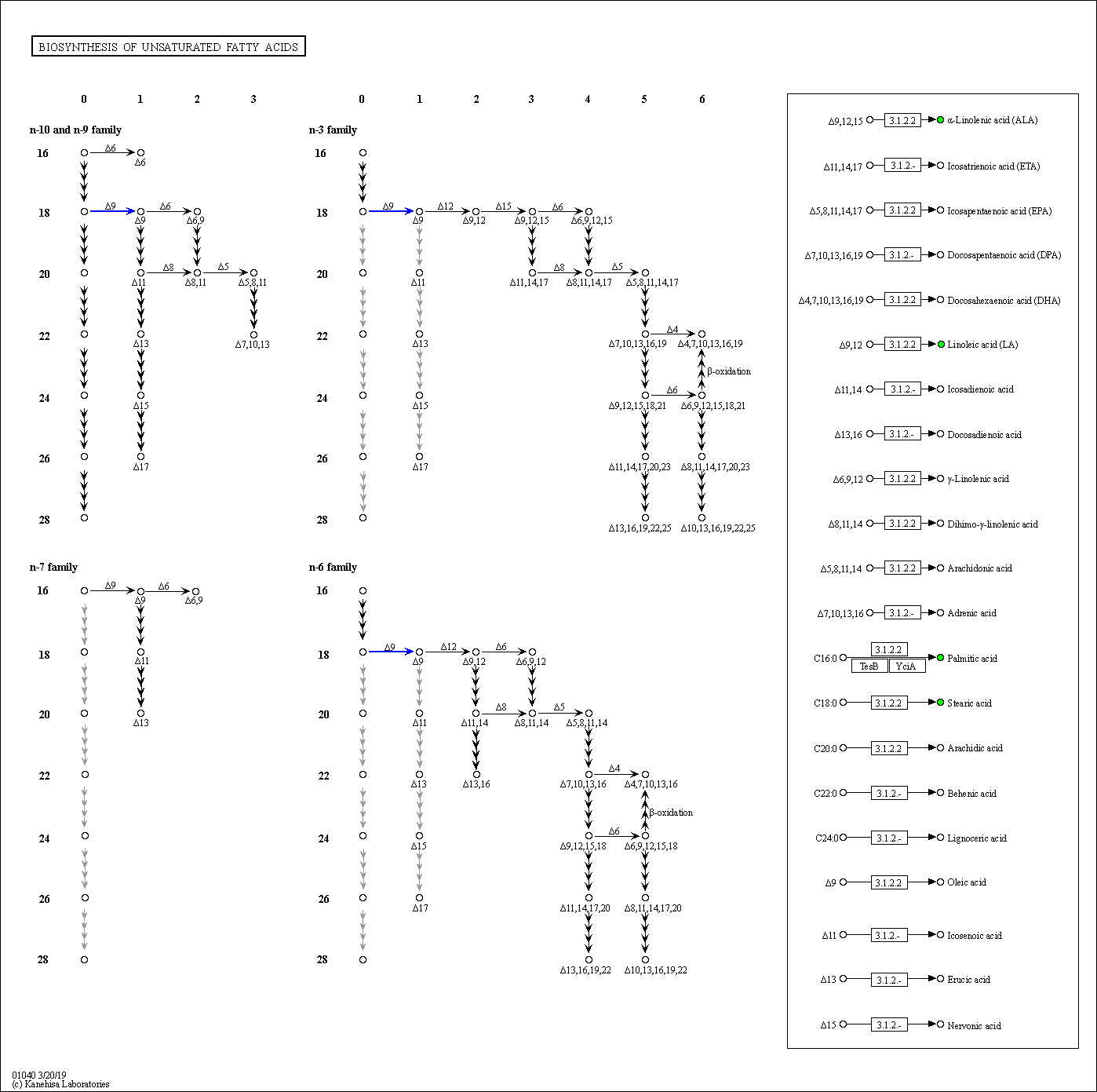


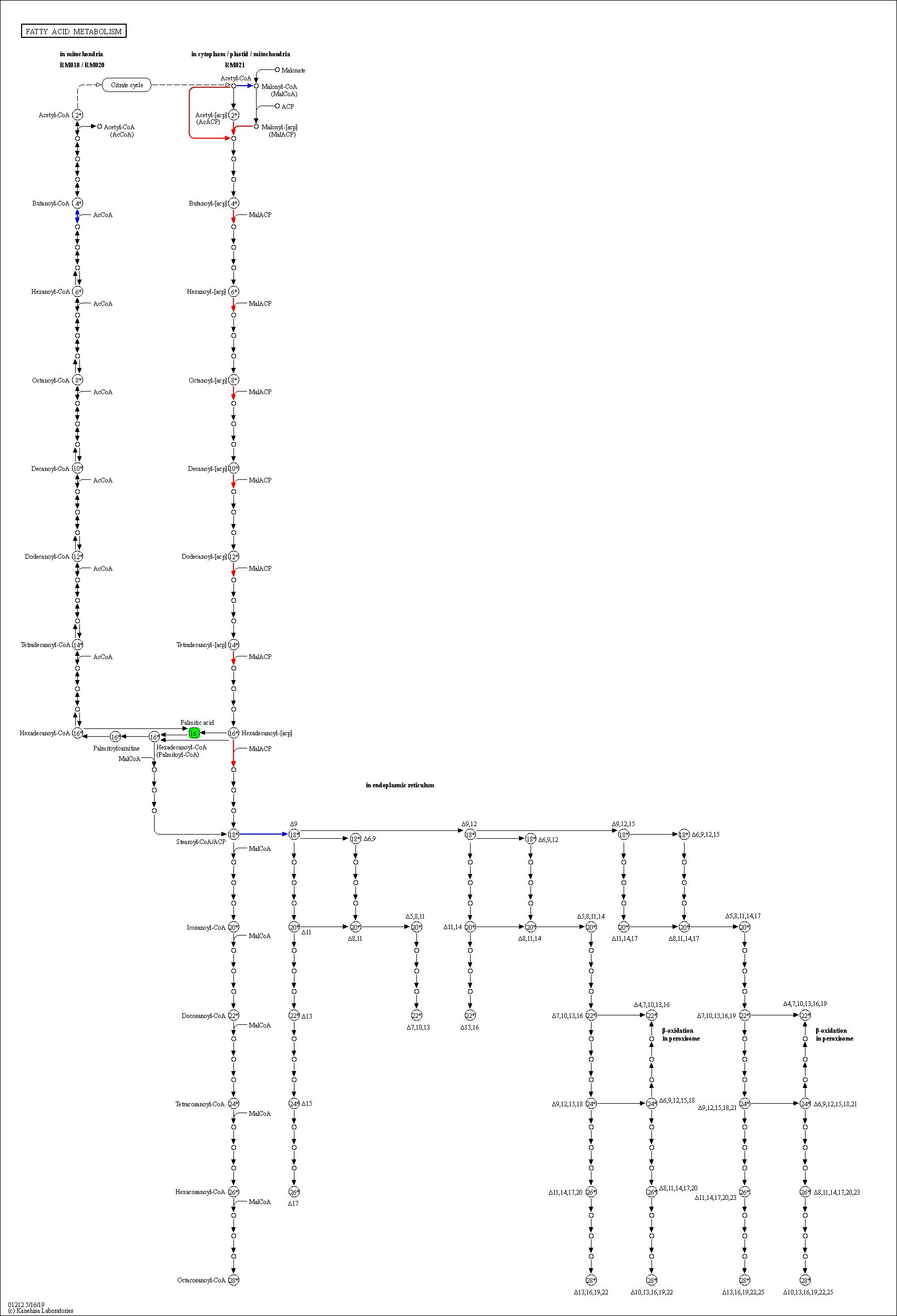

Supplement: Supplementary file 10 [file Data_Sheet_3.docx]
